# Blocking Lipid Uptake Pathways Does not Prevent Toxicity in Adipose Triglyceride Lipase (ATGL) Deficiency

**DOI:** 10.1016/j.jlr.2022.100274

**Published:** 2022-09-15

**Authors:** Jide Oluwadare, Ainara G. Cabodevilla, Ni-Huiping Son, Yunying Hu, Adam E. Mullick, Michael Verano, Jose O. Alemán, Ravichandran Ramasamy, Ira J. Goldberg

**Affiliations:** 1 Division of Endocrinology, Diabetes and Metabolism, Department of Medicine, New York University Grossman School of Medicine, New York, NY, USA; 2 Cardiovascular Drug Discovery, Ionis Pharmaceuticals Inc., Carlsbad, CA, USA

**Keywords:** CD36, dietary fat, lipid accumulation, lipotoxicity, storage diseases, LpL, lipid droplets, heart failure, myocardial lipid uptake, FA synthesis, AGC, automatic gain control, ASO, antisense oligonucleotide, ATGL, adipose triglyceride lipase, *cAtgl*^*−/−*^, cardiomyocyte-specific ATGL-deficient, CD36, cluster of differentiation 36, EF, ejection fraction, LD, lipid droplet, LpL, lipoprotein lipase, LV, left ventricle, LVID, LV internal dimension, 3-MA, 3-methyladenine, PI3K, phosphoinositide 3-kinase, PW, posterior wall, RNA-Seq, RNA sequencing, RRID, Research Resource Identifier, TC, total cholesterol, TG, triglyceride

## Abstract

Lipid accumulation in nonadipose tissues can cause lipotoxicity, leading to cell death and severe organ dysfunction. Adipose triglyceride lipase (ATGL) deficiency causes human neutral lipid storage disease and leads to cardiomyopathy; ATGL deficiency has no current treatment. One possible approach to alleviate this disorder has been to alter the diet and reduce the supply of dietary lipids and, hence, myocardial lipid uptake. However, in this study, when we supplied cardiac Atgl KO mice a low- or high-fat diet, we found that heart lipid accumulation, heart dysfunction, and death were not altered. We next deleted lipid uptake pathways in the ATGL-deficient mice through the generation of double KO mice also deficient in either cardiac lipoprotein lipase or cluster of differentiation 36, which is involved in an lipoprotein lipase-independent pathway for FA uptake in the heart. We show that neither deletion ameliorated ATGL-deficient heart dysfunction. Similarly, we determined that non-lipid-containing media did not prevent lipid accumulation by cultured myocytes; rather, the cells switched to increased de novo FA synthesis. Thus, we conclude that pathological storage of lipids in ATGL deficiency cannot be corrected by reducing heart lipid uptake.

Deficiency of adipose triglyceride (TG) lipase (ATGL), the rate-limiting enzyme for release of stored FAs within cells, leads to accumulation of TG in myocytes and in mice causes premature death because of heart failure (1, 2, 3). Humans with ATGL deficiency also have lipid accumulation in their skeletal muscles and with age develop cardiomyopathy (4, 5). There is no current treatment for this disorder. Although ATGL deficiency is often viewed as a model for lipotoxic cardiomyopathy, whether the primary cause of heart dysfunction is massive TG accumulation, inability to release lipid droplet (LD) stored TG, or mitochondrial dysfunction is unclear. Moreover, the driver of LD accumulation, whether lipid uptake or failed oxidation of intracellular lipids, is also unknown.

If the primary cause of heart dysfunction in ATGL deficiency is accumulation of circulating lipids, this disorder should be prevented if lipid uptake is reduced. Cardiomyocyte overexpression of PPARα, another mouse model of lipotoxic cardiomyopathy, was rescued by genetic deletion of cluster of differentiation 36 (CD36) and lipoprotein lipase (LpL) in the heart (6, 7). CD36 mediates transendothelial (8) and cardiomyocyte uptake (9) of long-chain FAs. LpL expression by cardiomyocytes is required for normal uptake of both chylomicron and VLDL-derived FAs (10). Another approach that has been advocated in humans is the use of a low fat medium-chain TG-enriched diet (11). Evidence that such a diet is beneficial is lacking.

We first tested whether the fat content of the diet altered heart pathology in cardiac ATGL-deficient mice. We then altered the major pathways of heart lipid uptake by deletion of LpL or CD36. Cultured cardiomyocytes were then studied to further elucidate the role of exogenous lipid in LD accumulation and survival in the context of ATGL deficiency. Our data show that modulating uptake of circulating lipids does not prevent cardiac dysfunction in ATGL deficiency.

## Materials and Methods

### Primers

**Table:** 

CD36 exon 4	Forward: AACACTGTGATTGTACCTG Reverse: TCAATAAGCATGTCTCCGAC
18S	Forward: CCAGTGGTCTTGGTGTGCTG Reverse: GGAGAACTCACGGAGGACGA
ATGL (Pnpla2)	Forward: GAGTGCAGTGTCCTTCACCA Reverse: ATCAGGCAGCCACTCCAAC
LpL	Forward: CGGCTTAGCTCAGTACTCAA Reverse: TCTAGGCAGAGAGCAGCAGA

### Antibodies

**Table:** 

LC3B antibody	Abcam; catalog no.: ab48394
Vinculin monoclonal antibody	Proteintech; catalog no.: 66305-1-Ig
Integrin beta1 antibody	Cell Signaling; catalog no.: 4706
p62/SQSTM1 antibody	Sigma-Aldrich; catalog no.: P0067-25UL
ATGL antibody #2138	Cell Signaling; catalog no.: 2138S
IRDye® 800CW donkey anti-goat IgG (H + L), 0.1 mg	Li-Cor; catalog no.: 925-32214
IRDye® 680RD donkey anti-mouse IgG (H + L), 0.1 mg	Li-Cor; catalog no.: 925-68072

### Inhibitors

**Table:** 

C75	Sigma-Aldrich; catalog no.: C5490
Cerulenin	MilliporeSigma; catalog no.: C2389
Platensimycin streptomyces	MilliporeSigma; catalog no.: 528244
Atglistatin ≥98% (HPLC)	Sigma-Aldrich; catalog no.: SML1075-5MG
Bafilomycin A1	Sigma-Aldrich; catalog no.: 19-148
3-Methyladenine (3-MA)	Fisher (Selleckchem); NC0737715 (catalog no.: S2767)
Rapamycin	Sigma-Aldrich; catalog no.: R0395
Lalistat 1	Tocris; catalog no.: 60-981-0

### Mouse studies

All procedures were conducted in conformity with the National Institutes of Health’s Public Health Service Policy on Humane Care and Use of Laboratory Animals and the National Research Council of the National Academy of Sciences’ Guide for the Care and Use of Laboratory Animals and were approved by the New York University Langone Medical Center Institutional Animal Care and Use Committee.

### Mice generation

*Atgl* floxed mice (The Jackson Laboratory; stock no.: 024278) were crossed with major histocompatibility complex-Cre expressing mice in laboratory to generate cardiomyocyte-specific ATGL-deficient (*cAtgl*^*−/−*^) mice. Genotype was confirmed by tail genomic DNA analysis. *cAtgl*^*−/−*^ mice were crossed with either *Lpl* floxed mice or *Cd36* floxed mice to generate double KO *cAtgl*^*−/−*^*Lpl*^*−/−*^ and *cAtgl*^*−/−*^*Cd36*^*−/−*^ mice, respectively. Genotype was confirmed by tail genomic DNA analysis.

### Mice maintenance, fasting, and harvesting

Mice were maintained under a constant light-dark cycle (Light from 7 AM to 7 PM) and received a standard diet (catalog no.: 5053; LabDiet), 60% kcal high-fat diet (catalog no.: D12492i; Research Diets), 90% kcal high-fat no carb diet (catalog no.: D1007080; Research Diets), or a 30% kcal sucrose and 40% kcal fat Western diet (catalog no.: 100244; Dyets). Mice were fasted overnight for ∼17 h for the CD36 antisense oligonucleotide (ASO) experiments or fasted for ∼5 h for all other experiments prior to sacrifice. After fasting, mice were flushed with PBS, and organs and tissues were harvested and immediately frozen on dry ice and stored at −80°C for future procedures.

### Measurement of plasma lipids and glucose

Mice were fasted for 5 h and then bled retro-orbitally under anesthesia (∼2% isoflurane), and the blood was spun at 10,000 *g* for 10 min to separate the plasma from the blood cells. Plasma was isolated from the aqueous layer. Plasma total cholesterol (TC), TG, and FFAs were measured enzymatically using TC (catalog no.: TR13421; Infinity, Thermo Fisher Scientific), TG (catalog no.: TR2242; Infinity, Thermo Fisher Scientific), and NEFA C (catalog no.: NC9517308-1; Wako, Fisher) kits. Glucose was measured after a 5 h fast. A blade was used to create a small incision on the tails of the mice, and a glucometer was used to measure the glucose of these mice.

### Cell culture conditions

H9c2(2-1) cells (ATCC CRL-1446; ATCC) were used for cell culture experiments and cultured according to the established guidelines. To deplete LDs, media supplemented with 10% FBS were removed from cells, and FBS-free DMEM was added for 24 h. C75 (40 μM) (12, 13), cerulenin (40 μM) (13, 14), and platensimycin streptomyces (20 μM) (13) were used to inhibit FASN. Atglistatin was used to inhibit ATGL (40 μM) (15). To examine the effect of various interventions on cell survival, AC16 cardiomyocyte-like cells were used. They were cultured according to the established guidelines. Then cells were incubated in media without FBS and the addition of various compounds (40 μM atglistatin and/or 5 mM 3-MA, 100 nM rapamycin). Survival was calculated using change in cells per field over time.

### Lipid extraction from tissues

To examine tissue lipids, 40–50 mg of heart tissue was homogenized using a mini bead beater. Lipids were extracted using methods modified from the study by Folch *et al.* (16). Lipids were dried using a nitrogen blower and solubilized in distilled water containing 2% Triton X. TG was measured using the Infinity Triglycerides kit.

### Microscopy for cell visualization

Tissue lipids were examined via histochemistry. About 20 to 40 mg of heart tissue was embedded using optimal cutting temperature compound and flash frozen. Embedded samples were sectioned into 8-micron thick sections and stained for nuclei using 4',6-diamidino-2-phenylindole (1:10,000 dilution) and then neutral lipids using the BODIPY green dye (1:1,000 dilution) (Thermo Fisher Scientific). Fluorescent images were captured using the Leica SP5 Confocal Microscope, and green fluorescent signal (LD number) was counted using the ImageJ software (U. S. National Institutes of Health). These counts were normalized to 4',6-diamidino-2-phenylindole-stained nuclei.

### Microscopy for gross heart visualization

Leica S9i microscope was used to capture gross images of excised hearts.

### Gene expression

To examine tissue gene expression, 20–40 mg of heart tissue was homogenized using a Biospec mini bead beater. Total RNA was isolated using a Pure Link micro-to-Midi total purification kit (Invitrogen) and reverse transcribed using ThermoScript RT-PCR Kit (Invitrogen). Quantitative real-time PCR using SYBR Green PCR Master Mix (Applied Biosystems) was used to examine gene expression.

### RNA sequencing

#### Sequencing

To examine tissue gene expression, 20–40 mg of heart tissue was homogenized using a Biospec mini bead beater. Total RNA was isolated using a Pure Link micro-to-Midi total purification kit and DNase treated. Total RNA (1 μg input per sample) was input by the Illumina Stranded Total RNA Ribozero Gold prep. All samples underwent 11 PCR cycles. Final libraries were run on the Agilent tapestation system to verify library base pair size and quantified with the high sensitivity dsDNA qubit kit (Invitrogen). Each library was normalized and pooled equally. Final normalized pool was sequenced on half an SP100 (1 lane) Illumina Novaseq flow cell, run as paired end 50 on the Illumina Novaseq 6000.

#### Analysis

RNA sequencing (RNA-Seq) differential expression analysis was performed for one lane of an SP100 paired-end 50 Illumina NovaSeq 6000 run. Per-read per-sample FASTQ files were generated using the bcl2fastq2 Conversion software (version 2.20) to convert per-cycle BCL base call files outputted by the sequencing instrument (RTA, version 3.4.4) into the FASTQ format. The alignment program, STAR (version 2.6.1d), was used for mapping reads of 13 samples to the mouse reference genome mm10, and the application Fastq Screen (version 0.13.0) was utilized to check for contaminants. The software, featureCounts (Subread package, version 1.6.3), was used to generate matrices of read counts for annotated genomic features. For differential gene statistical comparisons between groups of replicate samples contrasted by *Atgl*^*flox/flox*,^ c*Atgl*^*−/−*^, and c*Atgl*^*−/−*^*Lpl*^*−/−*^ conditions, the DESeq2 package (R, version 3.6.1) in the R statistical programming environment was utilized (17).

### Lipidomics

For lipidomics analysis, tissue was weighed to 20 mg and homogenized with 1 ml of methanol spiked with Avanti Lipid standards (Alabaster, AL). Lipids were extracted by taking 60 μl of homogenate in a 1:1:2 concentration of homogenate, LC-MS grade water, and chloroform. Homogenate solution was centrifuged to separate a top polar and a bottom nonpolar layer. Bottom layer chloroform was removed by vacuum centrifuge at 45^o^C for 30 min and resuspended in a 4:3:1 concentration of LC-MS grade isopropanol, acetonitrile, and water. Both top and bottom layers were run, and we focused on the nonpolar fraction for analysis.

### Metabolomics

#### Tissue metabolite extraction

A metabolite extraction was carried out on each sample based on a previously described method (18). Tissue processing began by precipitating proteins and other macromolecules within each sample by addition of extraction buffer of 80% methanol containing 500 nM isotopically labeled amino acid standards, whereas the controls were processed simultaneously. The extraction ratio was 10 mg of muscle sample per 1000 μl extraction buffer. The sample in extraction buffer mixture was subjected to a homogenization step with ∼100 μl zirconium disruption beads (0.5 mm, RPI) and homogenized for 10 min at 4°C in a BeadBlaster™ with a 30 s on, 30 s off pattern. The samples were subjected to three cycles of this homogenization step. Four stainless steel disruption beads (2.8 mm; Benchmark Scientific) were then added to each sample tube and subjected to two further cycles of the above homogenization step. The homogenized sample was centrifuged at 21,000 *g* for 3 min, and a fixed volume of the supernatant (450 μl) was transferred to a 1.5 ml microfuge tube for speed vacuum concentration; no heating was used. Muscle extracts were resolubilized in 50 μl of LC-MS grade water, and aliquots were transferred for metabolomics analysis.

#### LC-MS/MS with the hybrid metabolomics method

Samples were subjected to an LC-MS analysis to detect and quantify known peaks. The LC column was a Millipore™ ZIC-pHILIC (2.1 × 150 mm, 5 μm) coupled to a Dionex Ultimate 3000™ system, and the column oven temperature was set to 25°C for the gradient elution. A flow rate of 100 μl/min was used with the following buffers: A) 10 mM ammonium carbonate in water, pH 9.0 and B) neat acetonitrile. The gradient profile was as follows: 80–20% B (0–30 min), 20–80% B (30–31 min), and 80–80% B (31–42 min). Injection volume was set to 2 μl for all analyses (42 min total run time per injection).

MS analyses were carried out by coupling the LC system to a Thermo Q Exactive HF™ mass spectrometer operating in heated electrospray ionization mode. Method duration was 30 min with a polarity switching data-dependent Top 5 method for both positive and negative modes. Spray voltage for both positive and negative modes was 3.5 kV, and capillary temperature was set to 320^o^C with a sheath gas rate of 35, auxiliary gas of 10, and maximum spray current of 100 μA. The full MS scan for both polarities utilized 120,000 resolution with an automatic gain control (AGC) target of 3e6 and a maximum injection time of 100 ms, and the scan range was from *m*/*z* 67 to 1,000. Tandem MS spectra for both positive and negative modes used a resolution of 15,000, AGC target of 1e5, maximum injection time of 50 ms, isolation window of *m/z* 0.4, isolation offset of *m/z* 0.1, fixed first mass of *m/z* 50, and three-way multiplexed normalized collision energies of 10, 35, and 80. The minimum AGC target was 1e4 with an intensity threshold of 2e5. All data were acquired in profile mode.

#### Relative quantification of metabolites

The resulting Thermo™ RAW files were converted to a sqlite3 file containing the raw signal intensities for all samples. An in-house python script (Skeleton) was used for peak detection and quantification of all internal standards and sample peaks based on a previously established library of metabolite retention times and accurate masses adapted from the Whitehead Institute (19) and verified with authentic standards and/or high-resolution MS/MS spectral manually curated against the NIST14MS/MS and METLIN (2017) (20) tandem mass spectral libraries. Metabolite peaks were extracted based on the theoretical *m*/*z* of the expected ion type (e.g., [M + H]^+^), with a ±5 part-per-million tolerance, and a ±7.5 s peak apex retention time tolerance within an initial retention time search window of ±0.5 min across the study samples. The resulting data matrix of metabolite intensities for all samples and blank controls was processed with an in-house statistical pipeline Metabolyze, version 1.0, and final peak detection was calculated based on a signal-to-noise ratio of 3× compared with blank controls, with a floor of 10,000 (arbitrary units). For samples where the peak intensity was lower than the blank threshold, metabolites were annotated as not detected, and the threshold value was imputed for any statistical comparisons to enable an estimate of the fold change as applicable. The resulting blank corrected data matrix was then used for all groupwise comparisons, and *t*-tests were performed with the Python SciPy (version 1.1.0) (21) library to test for differences and generate statistics for downstream analyses. Any metabolite with *P* < 0.05 was considered significantly regulated (up or down).

### Endothelial cell isolation

Endothelial cells were isolated using the protocol adapted from the study by van Beijnum *et al.* (22). Heart tissue was minced and treated with a solution of 0.9 ml collagenase and 0.1 ml dispase per 100 mg of tissue. Then the tissue suspension was incubated for 30 min at 37°C, under continuous agitation. About 7.5 μl DNaseI solution per 1 ml cell suspension was added, and suspension was incubated for another 30 min in 37°C. About 0.5 ml of cold DMEM was then added for every 1 ml of collagenase/dispase solution to the tissue suspension. The suspension was sieved through a 100-μm cell strainer fitted on a 50-ml tube on ice to remove undigested cell clumps and separate the single cells, the filter rinsed with 2 ml cold DMEM, and the cells collected by centrifugation at 400 *g* for 5 min at room temperature. To remove red blood cells, the suspension was treated with red blood cell lysis buffer. For magnetic bead isolation, cell suspension was centrifuged at 300 *g* for 10 min, and the supernatant was completely aspirated. The final cell pellet was resuspended in 90 μl of protein extraction buffer, and an established protocol for Miltenyi Biotec CD31 microbead-based isolation was followed. To further validate the purity of microbead isolated cells, the *Cd31* gene expression of bound cells (elemental carbon fraction, CD31+) and nonbound cells (CD31−) was compared. We observed CD31 differential expression comparable to flow cytometry-sorted cells (CD31+).

### Western blotting

Protein was extracted from flash-frozen tissues through homogenization in RIPA buffer using minibeads. BSA protein assay kit (Thermo Fisher Scientific) was used to determine the concentration of proteins. Western blotting was performed using established protocols.

### Echocardiography

Echocardiograms were obtained using the Vevo2100 Ultrasound on mice under anesthesia (∼2% isoflurane) while temperature was maintained at 37°C with a heated pad and heat lamp. Parasternal long-axis and short-axis views of the heart were obtained. In the long-axis view, visualization of the apex, ascending aorta, and aortic reflex ensures proper orientation. The short-axis view was obtained by rotating the probe 90° after appropriately acquiring the long-axis view. Visualization of the circular contracting left ventricle (LV) as well as the papillary muscles ensured proper orientation. M-mode recordings were made from the midline of the LV in the short-axis view. Measurements used were obtained from the M-mode view. Measurements acquired include LV interventricular septal thicknesses, LV internal dimensions (LVIDs), and posterior wall (PW) thicknesses at diastole and systole. LV ejection fraction (EF), LV fractional shortening, and LV posterior wall thickening were calculated using the following formulas: EF (%) = 100 × [(LVIDd (3) − LVIDs (3))/LVIDd (3)]; fractional shortening (%) = 100 × [(LVIDd − LVIDs)/LVIDd]; and posterior wall thickening (%) = 100 ∗ [(PWs − PWd)/PWd].

### Statistical analysis

Statistical analyses were performed using GraphPad Prism (GraphPad Software, Inc), and all graphs are shown using standard deviation. Statistical significance when comparing two groups was determined by an unpaired *t*-test. Statistical significance when comparing three or more groups was determined by one-way ANOVA with Tukey multiple comparisons test. Statistical significance when comparing multiple unrelated parameters of multiple groups in the same analysis was determined by two-way ANOVA with Fisher’s least significant difference multiple comparisons test. Statistical significance when comparing multiple related parameters of multiple groups was determined by two-way ANOVA with Tukey correction for multiple comparisons.

## Results

### Effects of dietary carbohydrate on heart function and survival of ATGL-deficient mice

**Fig. 1:**
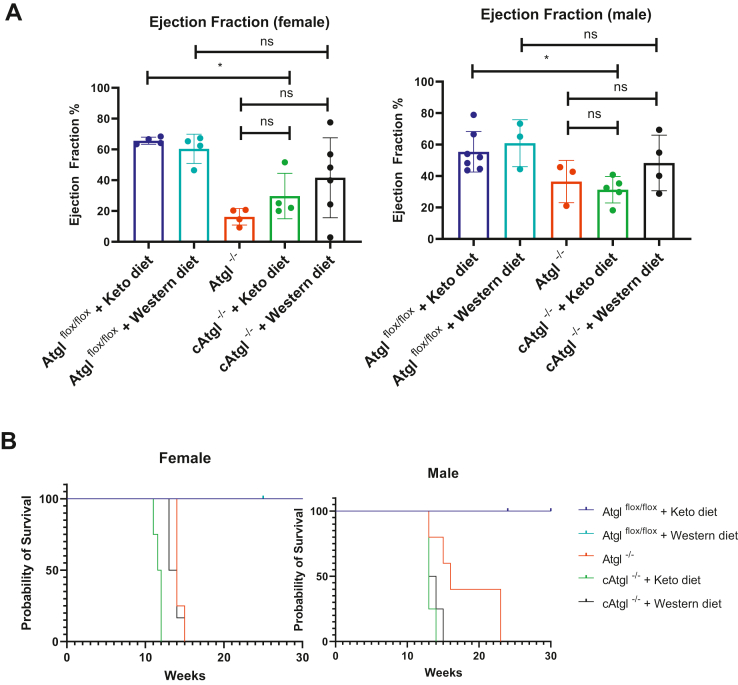
Treatment of *cAtgl*^*−/−*^ mice with ketogenic or Western diets. Five-week-old male and female mice were fed either a ketogenic, Western, or chow diet for 5 weeks and then examined for changes in EF and survival. A: Neither ketogenic diet nor Western diet significantly improved EF of male mice (*Atgl*^*flox/flox*^ + ketogenic diet [*n* = 4]; *Atgl*^*flox/flox*^ + Western diet [*n* = 4]; *cAtgl*^*−/−*^ + chow diet [*n* = 4]; *cAtgl*^*−/−*^ + ketogenic diet [*n* = 4]; *cAtgl*^*−/−*^ + Western diet [*n* = 6]) or female mice (*Atgl*^*flox/flox*^ + ketogenic diet [*n* = 7]; *Atgl*^*flox/flox*^ + Western diet [*n* = 3]; *cAtgl*^*−/−*^ + chow diet [*n* = 3]; *cAtgl*^*−/−*^ + ketogenic diet [*n* = 5]; *cAtgl*^*−/−*^ + Western diet [*n* = 4]). Statistical significance was determined by one-way ANOVA with Tukey multiple comparisons test; ∗*P* < 0.05, ∗∗*P* < 0.01, ∗∗∗*P* < 0.001, and ∗∗∗∗*P* < 0.0001. B: Survival was not improved either in male mice (*Atgl*^*flox/flox*^ + ketogenic diet [*n* = 6]; *Atgl*^*flox/flox*^ + Western diet [*n* = 4]; *cAtgl*^*−/−*^ + chow diet [*n* = 5]; *cAtgl*^*−/−*^ + ketogenic diet [*n* = 4]; *cAtgl*^*−/−*^ + Western diet [*n* = 4]) or female mice (*Atgl*^*flox/flox*^ + ketogenic diet [*n* = 5]; *Atgl*^*flox/flox*^ + Western diet [*n* = 4]; *cAtgl*^*−/−*^ + chow diet [*n* = 4]; *cAtgl*^*−/−*^ + ketogenic diet [*n* = 4]; *cAtgl*^*−/−*^ + Western diet [*n* = 6]). Survival data are from the mice shown in panel A with the addition of another cohort of mice. Statistical significance of Kaplan-Meier was determined by log-rank test with a *P* value cutoff of 0.05.

Because others have proposed the use of low-fat diets for humans with ATGL deficiency (11), we tested whether changing the dietary content of fat alters the phenotype of ATGL-deficient hearts. Although we had expected the higher fat-containing diets to worsen cardiomyopathy, neither a carbohydrate-poor diet (ketogenic diet) or carbohydrate-rich diet (Western diet) improved EF (Fig. 1A) or survival (Fig. 1B).

### LpL deficiency does not improve hearts of ATGL-deficient mice

**Fig. 2:**
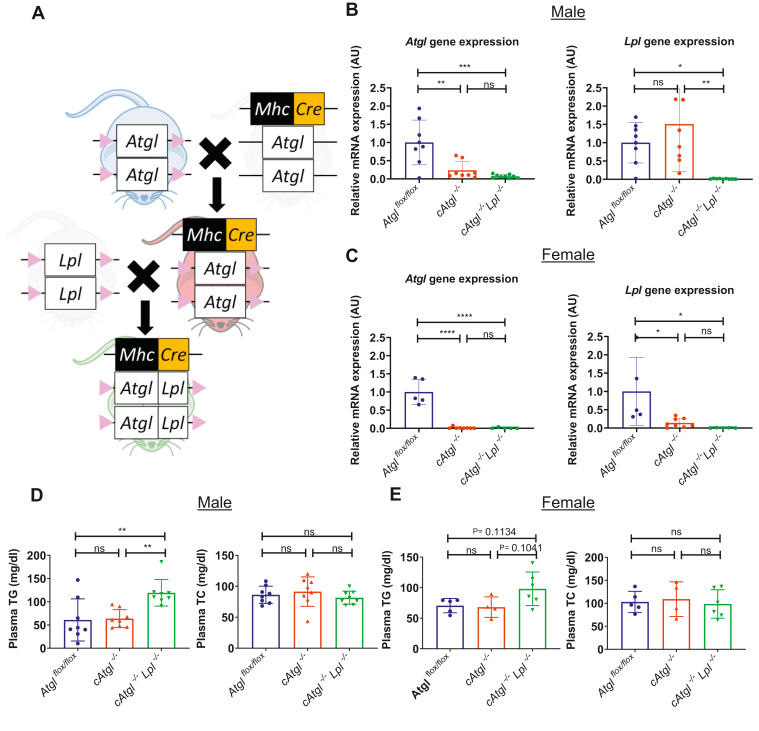
Creation of *cAtgl*^*−/−*^*Lpl*^*−/−*^ mice. A: Breeding of *Atgl*/*Lpl* cardiomyocyte-specific double KO mice from *Atgl*^*flox/flox*^ mice and major histocompatibility complex-Cre expressing mice with a C57BL/6 background. B: mRNA quantitative RT-PCR gene expression from homogenized whole heart in ∼10-week-old male *Atgl*^*flox/flox*^ (*n* = 8), *cAtgl*^*−/−*^ (*n* = 8), and *cAtgl*^*−/−*^*Lpl*^*−/−*^ mice (*n* = 9) as well as (C) female ∼10-week-old *Atgl*^*flox/flox*^ (*n* = 5), *cAtgl*^*−/−*^ (*n* = 8), and *cAtgl*^*−/−*^*Lpl*^*−/−*^ (*n* = 5) mice. They show decreased *Atgl* gene expression in male and female *cAtgl*^*−/−*^ and *cAtgl*^*−/−*^*Lpl*^*−/−*^ mice and decreased *Lpl* gene expression in male and female *cAtgl*^*−/−*^*Lpl*^*−/−*^ mice as well as female *cAtgl*^*−/−*^ mice. Data are corrected for 18S rRNA and normalized to *Atgl*^*flox/flox*^ controls. D: Plasma TG and TC of 10-week-old male mice (*Atgl*^*flox/flox*^ [*n* = 8]; *cAtgl*^*−/−*^ [*n* = 8]; and *cAtgl*^*−/−*^*Lpl*^*−/−*^ [*n* = 8]) and (E) female mice (*Atgl*^*flox/flox*^ [*n* = 5]; *cAtgl*^*−/−*^ [*n* = 4]; and *cAtgl*^*−/−*^*Lpl*^*−/−*^ [*n* = 6]) after a 5 h fast show increased TG in male *cAtgl*^*−/−*^*Lpl*^*−/−*^ mice. Statistical significance was determined by one-way ANOVA with Tukey multiple comparisons test; ∗*P* < 0.05, ∗∗*P* < 0.01, ∗∗∗*P* < 0.001, and ∗∗∗∗*P* < 0.0001.

To determine whether KO of LpL reduced heart dysfunction and premature death, we created mice with cardiac KO of both ATGL and LpL. First, we bred *Atgl*^*flox/flox*^ mice with major histocompatibility complex-Cre expressing mice to generate cardiomyocyte-specific *Atgl* deletion, *cAtgl*^*−/−*^ (Figs. 2A and S1A). Heart *Atgl* mRNA was decreased by over 80% in male and female mice. *Lpl* gene expression was reduced in female *cAtgl*^*−/−*^ mice but not in males (Fig. 2B, C), consistent with a previous report of reduced LpL activity in hearts of female muscle-specific ATGL-deficient mice (2). We next bred *cAtgl*^*−/−*^ mice with *Lpl*^*flox/flox*^ mice to generate cardiomyocyte-specific *cAtgl*^*−/−*^*Lpl*^*−/−*^ mice (Fig. 2A, S. Fig. 1A). As expected, cardiomyocyte-specific LpL deficiency increased circulating TG but not cholesterol levels (10) (Fig. 2D, E). TG levels were more dramatically increased in male *cAtgl*^*−/−*^*Lpl*^*−/−*^ mice, consistent with the difference in *Lpl* mRNA expression between male and female *cAtgl*^*−/−*^ mice.

**Fig. 3:**
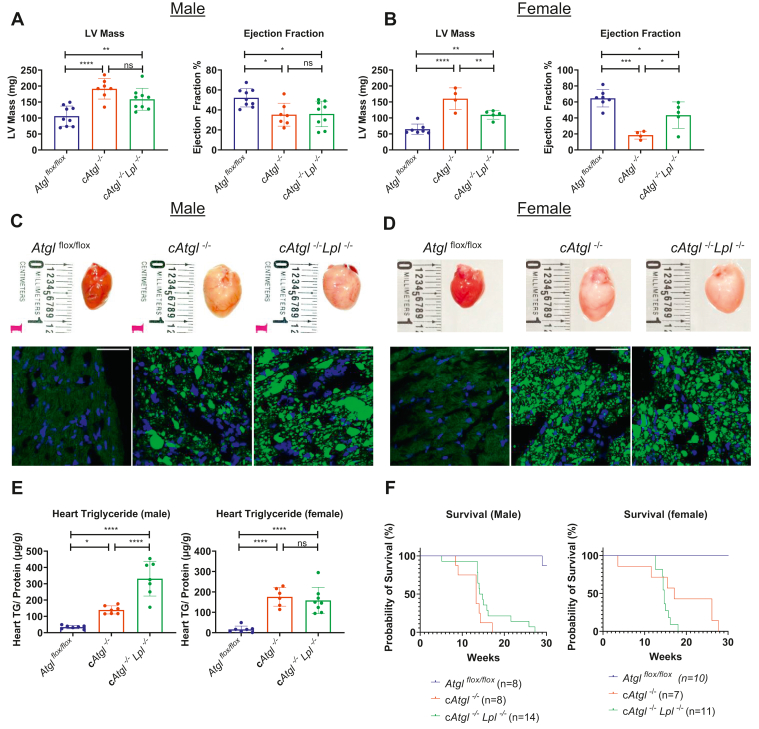
Effect of LpL deletion on *cAtgl*^*−/−*^ mice. A: Echocardiographic analysis of *cAtgl*^*−/−*^ mice showed increased LV mass and decreased EF that was not improved by LpL deletion in 10-week-old male (*Atgl*^*flox/flox*^ [*n* = 9]; *cAtgl*^*−/−*^ [*n* = 7]; and *cAtgl*^*−/−*^*Lpl*^*−/−*^ [*n* = 9]) but was normalized in (B) female mice (*Atgl*^*flox/flox*^ [*n* = 4]; *cAtgl*^*−/−*^ [*n* = 4]; and *cAtgl*^*−/−*^*Lpl*^*−/−*^ [*n* = 5]). C and D, upper panels: Images of hearts from 10-week-old *Atgl*^*flox/flox*^, *cAtgl*^*−/−*^, and *cAtgl*^*−/−*^*Lpl*^*−/−*^ male and female mice. Pale discoloration because of lipid accumulation in the *cAtgl*^*−/−*^ still present with LpL deletion in both male and female mice. Heart tissue sections from the three genotypes were stained with the neutral lipid stain BODIPY (green) and 4',6-diamidino-2-phenylindole (DAPI) (blue). The increased BODIPY staining in the *cAtgl*^*−/−*^ was not decreased by LpL deletion in male or female 10-week-old mice (C and D, lower panels). The scale bars represent 50 μm. E: Heart TG content in male mice (*Atgl*^*flox/flox*^ [*n* = 7]; *cAtgl*^*−/−*^ [*n* = 7]; *cAtgl*^*−/−*^*Lpl*^*−/−*^ [*n* = 7]) and female mice (*Atgl*^*flox/flox*^ [*n* = 7]; *cAtgl*^*−/−*^ [*n* = 6]; *cAtgl*^*−/−*^*Lpl*^*−/−*^ [*n* = 8]) show a similar pattern. Statistical significance was determined by one-way ANOVA with Tukey multiple comparisons test; ∗*P* < 0.05, ∗∗*P* < 0.01, ∗∗∗*P* < 0.001, and ∗∗∗∗*P* < 0.0001. F, left panel: Kaplan-Meier plot showing the cumulative survival of male *Atgl*^*flox/flox*^ (*n* = 8), *cAtgl*^*−/−*^ (*n* = 8), and *cAtgl*^*−/−*^*Lpl*^*−/−*^ (*n* = 14) mice over 30 weeks. F, right panel: Kaplan-Meier plot showing the cumulative survival of female *Atgl*^*flox/flox*^ (*n* = 10), *cAtgl*^*−/−*^ (*n* = 7), and *cAtgl*^*−/−*^*Lpl*^*−/−*^ (*n* = 11) mice over 30 weeks. LpL deletion did not improve survival of *cAtgl*^*−/−*^ mice.

Echocardiography at approximately 10 weeks after birth showed that ATGL deficiency increased heart mass in male and female mice (Fig. 3A, B and Table 1A, B). LV diameter and volume were decreased during the end of diastole but increased during late systole, which correlated with decreased EF (Table 1A). This could be due to fibrosis caused by ATGL deficiency as seen in *Atgl*^*−/−*^ and inducible c*Atgl*^*−/−*^ mice (1, 3). Despite their increased heart rates, cardiac output in these mice was reduced because of a decreased stroke volume. LV anterior and posterior wall thickness reflected the changes in LV diameter and volume (Table 1A).

**Table 1:** Echocardiography of *Atgl*^*flox/flox*^, *cAtgl*^*−/−*^, and *cAtgl*^*−/−*^*Lpl*^*−/−*^ mice

Male (A)	*Atgl*^*flox/flox*^			*cAtgl*^*−/−*^			*cAtgl*^*−/−*^*Lpl*^*−/−*^			
	Mean	SD	*N*	Mean	SD	*N*	Mean	SD	*N*	
Heart rate (BPM)	408	28	9	462	103	7	467	66	9	∗, #
Diameter; s (mm)	3.28	0.47	9	3.38	0.55	7	3.48	0.64	9	∗, #
Diameter; d (mm)	4.47	0.39	9	4.06	0.52	7	4.18	0.47	9	∗, #
Volume; s (μl)	45.05	15.61	9	48.87	17.70	7	52.98	22.85	9	∗, #
Volume; d (μl)	92.24	18.94	9	74.32	22.46	7	79.19	20.65	9	∗, #
Stroke volume (μl)	47.20	7.92	9	25.45	8.51	7	26.21	4.86	9	∗, #
EF (%)	52.15	8.63	9	35.28	10.60	7	36.01	12.08	9	∗, #
Fractional shortening (%)	26.85	5.48	9	16.90	5.85	7	17.33	6.30	9	∗, #
Cardiac output (ml/min)	19.25	3.46	9	11.23	3.44	7	12.15	2.42	9	∗, #
LV mass (mg)	132.39	37.13	9	239.12	37.07	7	198.23	40.42	9	∗, #
LV mass correct (mg)	105.91	29.70	9	191.29	29.65	7	158.58	32.34	9	∗, #
LVAW; s (mm)	1.17	0.16	9	1.59	0.39	7	1.39	0.14	9	∗, #
LVAW; d (mm)	0.77	0.15	9	1.27	0.29	7	1.14	0.16	9	∗, #
LVPW; s (mm)	0.99	0.20	9	1.55	0.28	7	1.23	0.20	9	∗, #
LVPW; d (mm)	0.73	0.18	9	1.32	0.24	7	1.06	0.15	9	∗, #

Female (B)	*Atgl*^*flox/flox*^			*cAtgl*^*−/−*^			*cAtgl*^*−/−*^*Lpl*^*−/−*^			
	Mean	SD	*N*	Mean	SD	*N*	Mean	SD	*N*	
Heart rate (BPM)	403	43	7	426	75	4	442	36	5	∗, #, %
Diameter; s (mm)	2.47	0.48	7	4.37	0.32	4	3.14	0.81	5	∗, #, %
Diameter; d (mm)	3.78	0.33	7	4.77	0.26	4	3.95	0.59	5	∗, #, %
Volume; s (μl)	23.17	10.81	7	87.06	15.24	4	43.52	28.31	5	∗, #, %
Volume; d (μl)	62.15	12.90	7	106.25	13.51	4	70.32	25.69	5	∗, #, %
Stroke volume (μl)	38.98	3.96	7	19.20	3.21	4	26.80	4.64	5	∗, #, %
EF (%)	64.62	10.08	7	18.51	4.31	4	43.49	14.83	5	∗, #, %
Fractional shortening (%)	35.28	7.48	7	8.37	2.02	4	21.59	7.94	5	∗, #, %
Cardiac output (ml/min)	15.70	2.15	7	8.11	1.91	4	11.70	1.35	5	∗, #, %
LV mass (mg)	81.06	18.50	7	200.28	36.68	4	137.49	15.41	5	∗, #, %
LV mass correct (mg)	64.85	14.80	7	160.22	29.34	4	109.99	12.33	5	∗, #, %
LVAW; s (mm)	1.14	0.11	7	1.15	0.27	4	1.17	0.26	5	∗, #, %
LVAW; d (mm)	0.69	0.11	7	0.87	0.13	4	0.97	0.17	5	∗, #, %
LVPW; s (mm)	0.87	0.14	7	1.13	0.24	4	1.06	0.13	5	∗, #, %
LVPW; d (mm)	0.60	0.11	7	1.05	0.28	4	0.86	0.11	5	∗, #, %

Measurements include heart rate, LV systolic diameter, LV diastolic diameter, LV systolic volume, LV diastolic volume, stroke volume, EF, fractional shortening, cardiac output, LV mass, LV mass correct by constant derived by Visualsonic Vevo 2100 ultrasound system to more accurately determine LV mass, LV anterior wall thickness during systole, LV anterior wall thickness during diastole, LV posterior wall thickness during systole and LV posterior wall thickness during diastole for male (A) and female (B) mice. ∗ = *Atgl*^*flox/flox*^ versus *cAtgl*^*−/−*^, *P* < 0.05, # = *Atgl*^*flox/flox*^ versus *cAtgl*^*−/−*^*Lpl*^*−/−*^, *P* < 0.05, % = *cAtgl*^*−/−*^ versus *cAtgl*^*−/−*^*Lpl*^*−/−*^, *P* < 0.05. Statistical significance was determined by two-way ANOVA with Fisher’s least significant difference multiple comparisons test.

Both male and female *cAtgl*^*−/−*^ hearts were pale and enlarged, and deletion of *Lpl* did not change the gross appearance (Fig. 3C, D, upper panels). Surprisingly, loss of LpL neither did reduce cardiomyocyte neutral lipid accumulation as shown by BODIPY 493/503 staining of neutral lipids in heart sections (Fig. 3C, D, lower panels, green) nor did reduce TG per milligram of heart protein (Fig. 3E). Heart TG levels averaged higher in males but not females with the double KO. The reasons for this are unclear but might indicate non-LpL pathways of lipid uptake, which have been described recently (23, 24).

All tissue-specific *Atgl* KO mice began to die at around 12 weeks. As has been shown previously (1), although some *Atgl*^*−/−*^ female mice survived longer (50% at 17 weeks) than their male counterparts (50% at 13 weeks), LpL deficiency did not improve overall survival of either male or female *cAtgl*^*−/−*^ mice as assessed using log-rank test analysis (Fig. 3F).

### Cardiomyocyte CD36 deficiency does not improve ATGL-deficient hearts

**Fig. 4:**
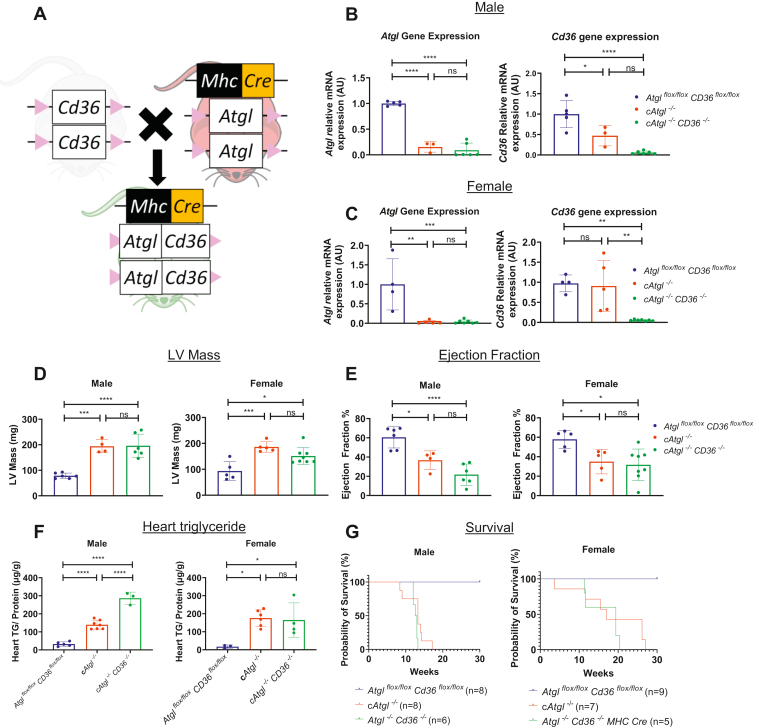
Creation of *cAtgl*^*−/−*^*Cd36*^*−/−*^ mice and characterization of effect of CD36 deletion on *cAtgl*^*−/−*^ mice. A: Breeding of *Atgl*/*Cd36* cardiomyocyte-specific double KO mice from *cAtgl*^*−/−*^ mice and *Cd36*^*flox/flox*^ mice with a C57BL/6 background. B: mRNA quantitative RT-PCR gene expression from homogenized whole heart in 10-week-old male mice (*Atgl*^*flox/flox*^ [*n* = 5]; *cAtgl*^*−/−*^ [*n* = 3]; and *cAtgl*^*−/−*^*Cd36*^*−/−*^ [*n* = 6]) and (C) female mice (*Atgl*^*flox/flox*^ [*n* = 4]; *cAtgl*^*−/−*^ [*n* = 5]; and *cAtgl*^*−/−*^*Cd36*^*−/−*^ [*n* = 7]) show decreased *Atgl* gene expression in male and female *cAtgl*^*−/−*^ and *cAtgl*^*−/−*^*Cd36*^*−/−*^ mice; and decreased *Cd36* gene expression in male and female *cAtgl*^*−/−*^*Cd36*^*−/−*^ mice. Data are corrected for 18S rRNA and normalized to *Atgl*^*flox/flox*^*Cd36*^*flox/flox*^ controls. D and E: Echocardiographic analysis of *cAtgl*^*−/−*^ mice showed increased LV mass (D) and decreased EF (E) that was not improved by CD36 deletion in 10-week-old male mice (*Atgl*^*flox/flox*^ [*n* = 6]; *cAtgl*^*−/−*^ [*n* = 4]; and *cAtgl*^*−/−*^*Cd36*^*−/−*^ [*n* = 6]) or female mice (*Atgl*^*flox/flox*^ [*n* = 5]; *cAtgl*^*−/−*^ [*n* = 5]; and *cAtgl*^*−/−*^*Cd36*^*−/−*^ [*n* = 8]). F: Increased heart TG content in male (*n* = 3–7) and female (*n* = 3–6) mice was not reduced by CD36 inhibition. G, left panel: Kaplan-Meier plot showing the cumulative survival of male *Atgl*^*flox/flox*^*Cd36*^*flox/flox*^ (*n* = 8), *cAtgl*^*−/−*^ (*n* = 8), and *cAtgl*^*−/−*^*Cd36*^*−/−*^ (*n* = 6) mice over 30 weeks. G, right panel: Kaplan-Meier plot showing the cumulative survival of female *Atgl*^*flox/flox*^*Cd36*^*flox/flox*^ (*n* = 9), *cAtgl*^*−/−*^ (*n* = 7), and *cAtgl*^*−/−*^*Cd36*^*−/−*^ (*n* = 5) mice over 30 weeks. CD36 deletion did not improve survival of *cAtgl*^*−/−*^ mice. (Note: TG data and survival data of *cAtgl*^*−/−*^ mice same as for Figure 2. TG isolation and quantification of *Atgl*^*flox/flox*^, *cAtgl*^*−/−*^, *cAtgl*^*−/−*^*Lpl*^*−/−*^, *Atgl*^*flox/flox*^*Cd36*^*flox/flox*^, and *cAtgl*^*−/−*^*Cd36*^*−/−*^ performed simultaneously) Statistical significance was determined by one-way ANOVA with Tukey multiple comparisons test; ∗*P* < 0.05, ∗∗*P* < 0.01, ∗∗∗*P* < 0.001, and ∗∗∗∗*P* < 0.0001.

CD36 mediates an alternative route of FA uptake into the heart that is independent of LpL. We next tested whether CD36 inhibition improves the heart function and survival of *cAtgl*^*−/−*^ mice. Data from our laboratory (8) and others (25, 26) have shown that cardiomyocyte CD36 inhibition reduces heart LDs. To create a double KO of ATGL and CD36 in cardiomyocytes, we crossed *cAtgl*^*−/−*^ mice with *Cd36*^*flox/flox*^ mice (Figs. 4A and S1B). *Atgl* and *Cd36* mRNAs were significantly decreased in both male and female mice (Fig. 4B, C).

Echocardiography at 9–11 weeks showed that ATGL deficiency increased heart mass in male and female mice (Fig. 4D and Table 2A, B). EF was not improved by CD36 deficiency (Fig. 4E and Table 2A, B). Like with LpL deficiency, deletion of CD36 led to increased TG content in hearts from males (Fig. 4F). In neither males nor females was loss of CD36 associated with changes in survival (Fig. 4G).

**Table 2:** Echocardiography of *Atgl*^*flox/flox*^*Cd36*^*flox/flox*^, *cAtgl*^*−/−*^, and *cAtgl*^*−/−*^*Cd36*^*−/−*^ mice

Male (A)	*Atgl*^*flox/flox*^*Cd36*^*flox/flox*^			*cAtgl*^*−/−*^			*cAtgl*^*−/−*^*Cd36*^*−/−*^			
	Mean	SD	*N*	Mean	SD	*N*	Mean	SD	*N*	
Heart rate (BPM)	458	38	6	518	73	4	410	137	6	∗, #
Diameter; s (mm)	2.74	0.32	6	3.68	0.32	4	4.10	0.45	6	∗, #
Diameter; d (mm)	4.05	0.12	6	4.47	0.23	4	4.55	0.33	6	∗, #
Volume; s (μl)	28.71	8.40	6	58.20	12.32	4	75.53	19.71	6	∗, #
Volume; d (μl)	72.21	5.11	6	91.45	10.91	4	95.55	17.10	6	∗, #
Stroke volume (μl)	43.50	7.14	6	33.25	7.95	4	20.02	7.97	6	∗, #
EF (%)	60.48	10.10	6	36.70	8.41	4	21.75	10.06	6	∗, #
Fractional shortening (%)	32.39	6.91	6	17.71	4.42	4	10.05	4.85	6	∗, #
Cardiac output (ml/min)	20.14	4.59	6	16.68	2.24	4	8.30	4.94	6	∗, #
LV mass (mg)	98.25	12.13	6	242.77	27.90	4	245.16	52.08	6	∗, #
LV mass correct (mg)	78.60	9.71	6	194.21	22.32	4	196.13	41.66	6	∗, #
LVAW; s (mm)	1.09	0.16	6	1.61	0.23	4	1.44	0.23	6	∗, #
LVAW; d (mm)	0.73	0.07	6	1.27	0.23	4	1.25	0.18	6	∗, #
LVPW; s (mm)	0.92	0.16	6	1.32	0.09	4	1.26	0.07	6	∗, #
LVPW; d (mm)	0.64	0.09	6	1.09	0.09	4	1.07	0.10	6	∗, #

Female (B)	*Atgl*^*flox/flox*^*Cd36*^*flox/flox*^			*cAtgl*^*−/−*^			*cAtgl*^*−/−*^*Cd36*^*−/−*^			
	Mean	SD	*N*	Mean	SD	*N*	Mean	SD	*N*	
Heart rate (BPM)	454	40	5	510	46	5	491	56	8	∗, #, %
Diameter; s (mm)	2.86	0.37	5	3.63	0.48	5	3.39	0.69	8	∗, #, %
Diameter; d (mm)	4.10	0.25	5	4.35	0.32	5	3.96	0.52	8	∗, #, %
Volume; s (μl)	32.15	10.53	5	57.17	17.91	5	50.14	24.14	8	∗, #, %
Volume; d (μl)	74.89	11.12	5	86.12	14.32	5	70.14	22.42	8	∗, #, %
Stroke volume (μl)	42.74	4.97	5	28.96	8.10	5	20.00	7.59	8	∗, #, %
EF (%)	57.88	8.27	5	34.99	11.33	5	31.70	15.12	8	∗, #, %
Fractional shortening (%)	30.44	5.44	5	16.82	5.84	5	15.12	7.67	8	∗, #, %
Cardiac output (ml/min)	19.34	2.41	5	14.75	4.35	5	9.87	3.84	8	∗, #, %
LV mass (mg)	116.79	40.41	5	232.98	22.27	5	189.12	37.51	8	∗, #, %
LV mass correct (mg)	93.43	32.33	5	186.39	17.82	5	151.29	30.01	8	∗, #, %
LVAW; s (mm)	1.18	0.28	5	1.58	0.15	5	1.40	0.19	8	∗, #, %
LVAW; d (mm)	0.76	0.15	5	1.22	0.19	5	1.12	0.12	8	∗, #, %
LVPW; s (mm)	1.05	0.37	5	1.32	0.07	5	1.27	0.13	8	∗, #, %
LVPW; d (mm)	0.75	0.26	5	1.15	0.15	5	1.13	0.10	8	∗, #, %

Measurements include heart rate, LV systolic diameter, LV diastolic diameter, LV systolic volume, LV diastolic volume, stroke volume, EF, fractional shortening, cardiac output, LV mass, LV mass correct by constant derived by Visualsonic Vevo 2100 ultrasound system to more accurately determine LV mass, LV anterior wall thickness during systole, LV anterior wall thickness during diastole, LV posterior wall thickness during systole, and LV posterior wall thickness during diastole for male (A) and female (B) mice. ∗ = *Atgl*^*flox/flox*^*Cd36*^*flox/flox*^ versus *cAtgl*^*−/−*^, *P* < 0.05, # = *Atgl*^*flox/flox*^*Cd36*^*flox/flox*^ versus *cAtgl*^*−/−*^*Cd36*^*−/−*^, *P* < 0.05, % = *cAtgl*^*−/−*^ versus *cAtgl*^*−/−*^*Cd36*^*−/−*^, *P* < 0.05. Statistical significance was determined by two-way ANOVA with Fisher’s least significant difference multiple comparisons test.

### CD36 ASO decreases lipid accumulation in overnight fasted and but not ATGL-deficient hearts

**Fig. 5:**
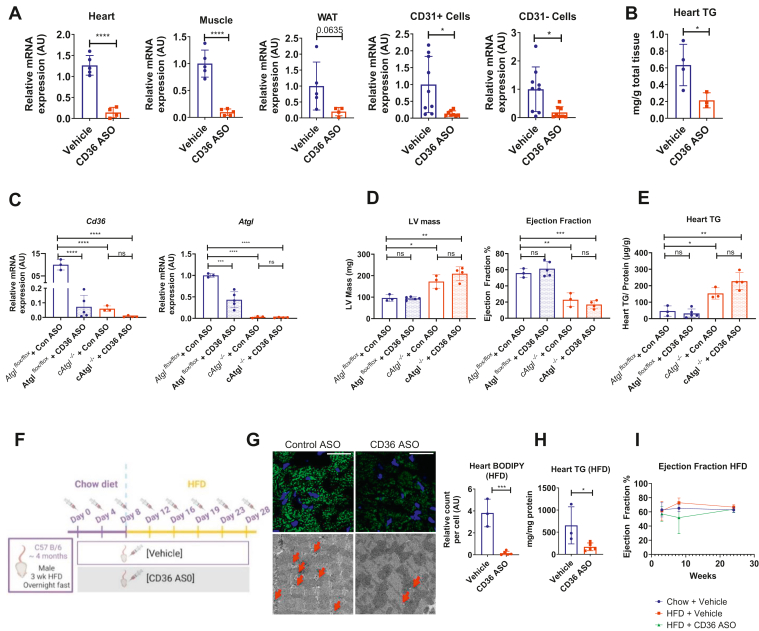
CD36 ASO treatment. A: Overnight (≈17 h) fasted C57B6 mice were injected with PBS (vehicle) or CD36 ASO (50 mg/kg body weight). ASO significantly decreased *Cd36* gene expression in whole heart (vehicle [*n* = 5], *Cd36* ASO [*n* = 4]), quadriceps (vehicle [*n* = 5], *Cd36* ASO [*n* = 5]), and inguinal white adipose (vehicle [*n* = 5], *Cd36* ASO [*n* = 4]) as well as in CD31+ (vehicle [*n* = 9], *Cd36* ASO [*n* = 8]) and CD31− cells (vehicle [*n* = 9], *Cd36* ASO [*n* = 8]) isolated from collagenase-disassociated hearts. B: Heart TG was decreased with CD36 ASO treatment (vehicle [*n* = 4], *Cd36* ASO [*n* = 3]). C: Experimental protocol for CD36 ASO treatment of HFD-fed animals. D and E: CD36 ASO significantly decreased heart TG content as shown in BODIPY-stained heart sections (D, top panels, green). The scale bars represent 25 μm (vehicle [*n* = 3], *Cd36* ASO [*n* = 5]), EM imaging of LDs (D, lower panels). The scale bars represent 2 μm, and whole-tissue TG quantification (E) (vehicle [*n* = 3], *Cd36* ASO [*n* = 5]). Statistical significance was determined by unpaired *t* test; ∗*P* < 0.05, ∗∗*P* < 0.01, ∗∗∗*P* < 0.001, and ∗∗∗∗*P* < 0.0001. F: EF as measured by echocardiography in control mice (chow-fed C57BL/6 mice treated with vehicle), and HFD-fed mice treated with vehicle or CD36 ASO ∗ = Chow + vehicle versus HFD + vehicle, *P* < 0.05, # = Chow + vehicle versus HFD + CD36 ASO, *P* < 0.05, % = HFD + vehicle versus HFD + CD36 ASO, *P* < 0.05. Statistical significance was determined by two-way ANOVA with Tukey correction for multiple comparisons. G: *Atgl* and *Cd36* gene expression in *Atgl*^*flox/flox*^ and *cAtgl*^*−/−*^ treated with either the control ASO or the CD36 ASO in male mice (*Atgl*^*flox/flox*^ + Con ASO [*n* = 3]; *Atgl*^*flox/flox*^ + CD36 ASO [*n* = 5]; *cAtgl*^*−/−*^ + Con ASO [*n* = 3]; *cAtgl*^*−/−*^ + CD36 ASO [*n* = 4]). *Atgl* gene expression was reduced in *cAtgl*^*−/−*^ mice as well as CD36 ASO-treated mice. The CD36 ASO reduced *Cd36* gene expression in the treated mice. ATGL deletion also led to decreased gene expression. H: Echocardiography analysis of changes in LV mass and EF in the four treatment groups of male mice shows increased LV mass and decreased EF in *cAtgl*^*−/−*^ mice that is not improved by the CD36 ASO (*Atgl*^*flox/flox*^ + Con ASO [*n* = 3]; *Atgl*^*flox/flox*^ + CD36 ASO [*n* = 5]; *cAtgl*^*−/−*^ + Con ASO [*n* = 3]; *cAtgl*^*−/−*^ + CD36 ASO [*n* = 4]). I: Heart TG changes from male mice of the four treatment groups (*Atgl*^*flox/flox*^ + Con ASO [*n* = 3]; *Atgl*^*flox/flox*^ + CD36 ASO [*n* = 5]; *cAtgl*^*−/−*^ + Con ASO [*n* = 3]; *cAtgl*^*−/−*^ + CD36 ASO [*n* = 4]) show that the CD36 ASO does not reduce TG accumulation. Statistical significance was determined by one-way ANOVA with Tukey multiple comparisons test; ∗*P* < 0.05, ∗∗*P* < 0.01, ∗∗∗*P* < 0.001, and ∗∗∗∗*P* < 0.0001.

Heart uptake of FAs may be more reliant on CD36 expression by endothelial cells than cardiomyocytes (8). Therefore, we assessed the effect of a global knockdown of *Cd36* using ASOs. With control ASO, CD36 expression as expected was lower in ATGL KO hearts, as CD36 is downstream of PPAR activation that requires ATGL. But this reduction was even greater with specific ASO; *Cd36* mRNA decreased >90% in the heart, quadriceps, and inguinal adipose of mice treated with CD36 ASO (Fig. 5A). There was a similar reduction both in CD31 positive (endothelial cells) and negative cells isolated from the cell suspension of collagenase-dissociated hearts (Fig. 5A). C57BL/6 mice fasted overnight develop LDs and increased TG content in various organs (27). In the heart, CD36 deficiency in either endothelial cells or cardiomyocytes prevents this accumulation (8). CD36 ASO similarly decreased heart TG content during fasting (Fig. 5B).

To assess whether systemic CD36 inhibition would reduce heart lipid accumulation and improve cardiac dysfunction in ATGL-deficient mice, we treated *Atgl*^*flox/flox*^ and *cAtgl*^*−/−*^ mice with either the CD36 ASO or a scrambled control for 5 weeks. The ASO treatment decreased *Cd36* gene expression (Fig. 5C, left panel) and protein levels (supplemental Fig. S2A) and as expected *Atgl* expression in *Atgl*^*flox/flox*^ mice was decreased by the CD36 ASO because of the effect of CD36 inhibition in cardiomyocytes on PPARα-regulated genes (7, 8) (Fig. 5C, right panel).

### CD36 ASO does not improve hearts of ATGL- and ATGL/LpL-deficient mice

The CD36 ASO treatment did not significantly improve heart discoloration (not shown), LV mass and EF (Fig. 5D and Table 3), or reduce TG accumulation (Fig. 5E) in male mice. We observed a similar trend in female mice (supplemental Fig. S2B–D and Table 3B). We then treated *cAtgl*^*−/−*^*Lpl*^*−/−*^ mice with this ASO to determine if CD36 compensated for loss of LpL. But even with loss of both pathways involved in lipid uptake, survival of neither male mice nor female mice was improved (supplemental Fig. S3A, B).

**Table 3:** Echocardiography of *Atgl*^*flox/floxx*^, *cAtgl*^*−/−*^ mice treated with either a control ASO or CD36 ASO

Male (A)	*Atgl*^*flox/flox*^*+* CON ASO			*Atgl*^*flox/flox*^*+* CD36 ASO			*cAtgl*^*−/−*^ + CON ASO			*cAtgl*^*−/−*^*+* CD36 ASO			
	Mean	SD	*N*	Mean	SD	*N*	Mean	SD	*N*	Mean	SD	*N*	
Heart rate (BPM)	448	42	3	437	42	5	514	55	3	512	30	4	#, %
Diameter; s (mm)	3.18	0.31	3	2.63	0.49	5	3.76	0.37	3	4.50	0.52	4	
Diameter; d (mm)	4.48	0.26	3	3.88	0.44	5	4.19	0.27	3	4.86	0.51	4	
Volume; s (μl)	41.04	9.26	3	26.91	12.37	5	61.25	14.75	3	94.09	23.38	4	%, @
Volume; d (μl)	91.76	12.37	3	66.55	18.51	5	78.50	12.21	3	112.71	25.33	4	∗, %, @
Stroke volume (μl)	50.72	3.64	3	39.64	7.17	5	17.26	3.91	3	18.62	5.59	4	#, %
EF (%)	55.86	4.54	3	61.32	8.23	5	22.85	6.96	3	16.95	4.35	4	#, %
Fractional shortening (%)	29.06	2.89	3	32.70	5.77	5	10.40	3.34	3	7.66	2.04	4	
Cardiac output (ml/min)	22.58	0.96	3	17.31	3.54	5	8.67	1.04	3	9.71	3.53	4	
LV mass (mg)	120.01	15.87	3	117.48	8.31	5	215.86	31.82	3	261.33	34.71	4	#, %, @
LV mass correct (mg)	96.01	12.69	3	93.98	6.65	5	172.69	25.46	3	209.07	27.77	4	#, %, @
LVAW; s (mm)	1.25	0.18	3	1.46	0.13	5	1.35	0.11	3	1.29	0.15	4	
LVAW; d (mm)	0.79	0.13	3	0.91	0.11	5	1.21	0.07	3	1.11	0.15	4	
LVPW; s (mm)	0.83	0.07	3	1.00	0.18	5	1.42	0.25	3	1.36	0.10	4	
LVPW; d (mm)	0.62	0.02	3	0.76	0.15	5	1.13	0.17	3	1.16	0.15	4	

Female (B)	*Atgl*^*flox/flox*^*+* CON ASO			*Atgl*^*flox/flox*^*+* CD36 ASO			*cAtgl*^*−/−*^ + CON ASO			*cAtgl*^*−/−*^*+* CD36 ASO			
	Mean	SD	*N*	Mean	SD	*N*	Mean	SD	*N*	Mean	SD	*N*	
Heart rate (BPM)	465	33	5	484	60	5	486	52	3	513	66	3	#, %
Diameter; s (mm)	3.15	0.57	5	2.80	0.56	5	3.49	0.59	3	3.10	0.91	3	
Diameter; d (mm)	4.31	0.50	5	3.98	0.38	5	4.22	0.28	3	3.85	0.65	3	
Volume; s (μl)	41.54	19.19	5	31.61	13.39	5	52.74	19.30	3	43.20	29.29	3	
Volume; d (μl)	85.45	24.69	5	70.37	15.44	5	79.89	12.03	3	66.87	27.79	3	
Stroke volume (μl)	43.91	6.30	5	38.76	5.60	5	27.15	10.15	3	23.67	6.25	3	
EF (%)	53.38	7.73	5	57.21	12.13	5	36.06	17.61	3	41.44	19.92	3	
Fractional shortening (%)	27.47	4.68	5	30.32	8.69	5	17.81	9.68	3	21.04	11.89	3	
Cardiac output (ml/min)	20.47	3.48	5	18.94	4.34	5	13.03	5.04	3	11.80	1.97	3	
LV mass (mg)	161.21	29.60	5	114.09	29.82	5	183.86	46.80	3	123.11	24.89	3	∗, %, #
LV mass correct (mg)	128.96	23.68	5	91.27	23.86	5	147.09	37.44	3	98.49	19.92	3	∗, %, #
LVAW; s (mm)	1.37	0.34	5	1.30	0.15	5	1.54	0.20	3	1.15	0.21	3	
LVAW; d (mm)	0.91	0.24	5	0.74	0.06	5	1.27	0.16	3	0.88	0.13	3	
LVPW; s (mm)	1.27	0.38	5	1.04	0.30	5	1.08	0.27	3	1.30	0.24	3	
LVPW; d (mm)	0.93	0.25	5	0.80	0.19	5	0.77	0.17	3	0.85	0.27	3	

Measurements for male (A) and female (B) mice include heart rate, LV systolic diameter, LV diastolic diameter, LV systolic volume, LV diastolic volume, stroke volume, EF, fractional shortening, cardiac output, LV mass, LV mass correct by constant derived by Visualsonic Vevo 2100 ultrasound system to more accurately determine LV mass, LV anterior wall thickness during systole, LV anterior wall thickness during diastole, LV posterior wall thickness during systole, and LV posterior wall thickness during diastole. ∗ = *Atgl*^*flox/flox*^*+* CON ASO versus *Atgl*^*flox/flox*^*+ CD36 ASO*, *P* < 0.05, # = *Atgl*^*flox/flox*^*+* CON ASO versus *cAtgl*^*−/−*^*+* CON ASO, *P* < 0.05, % = *Atgl*^*flox/flox*^*+* CON ASO versus *cAtgl*^*−/−*^*+ CD36 ASO*, *P* < 0.05, @ = *Atgl*^*−/−*^*+* CON ASO versus *cAtgl*^*−/−*^*+ CD36 ASO*, *P* < 0.05. Statistical significance was determined by two-way ANOVA with Fisher’s least significant difference multiple comparisons test.

### CD36 deficiency reduced high-fat diet cardiac lipid accumulation

To confirm that CD36 ASO affected heart lipid metabolism, we treated mice eating a 60% high-fat diet fed with this ASO every 4 days for 4 weeks. Circulating lipids in these mice were similar to those in untreated mice (TG: 77.20 ± 24.02 mg/dl control ASO, 82.26 ± 35.15 CD36 ASO; TC: 79.50 ± 21.84 control ASO, 82.86 ± 20.59 mg/dl CD36 ASO). Although within this period, we found no change in cardiac function, the ASO reduced LDs and TG in the hearts (Fig. 5F–I).

### Gene and metabolite changes in ATGL KO mice

**Fig. 6:**
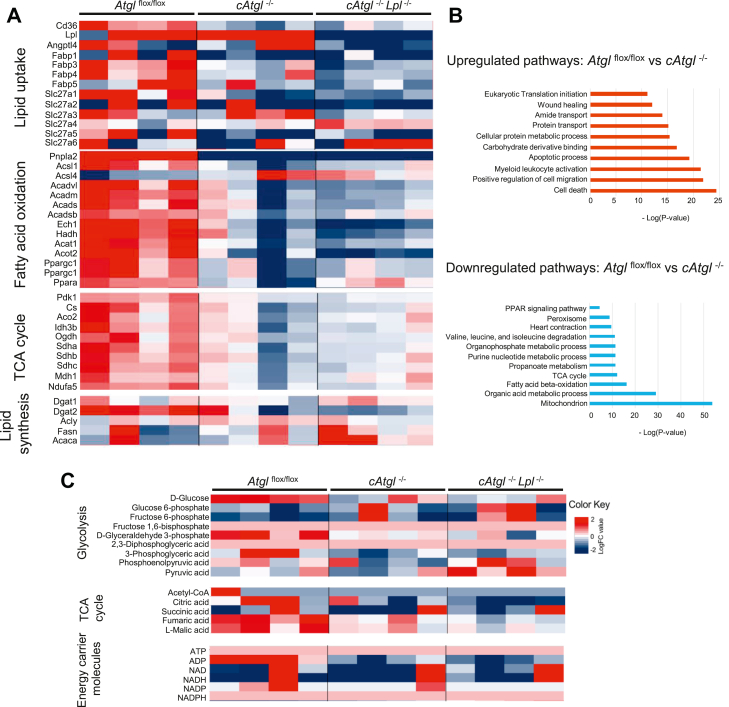
Heart tissue mRNA expression and metabolomics changes in *cAtgl*^*−/−*^ and *cAtgl*^*−/−*^*Lpl*^*−/−*^ mice. A: RNA-Seq analysis of changes between 6-week-old *Atgl*^*flox/flox*^, *cAtgl*^*−/−*^, and *cAtgl*^*−/−*^*Lpl*^*−/−*^ mice in expression of genes associated with lipid uptake, FA oxidation, TCA cycle, and lipid synthesis. B: Pathway analysis was performed to identify the most upregulated and downregulated pathways. C: Metabolomic analysis of changes in glycolysis intermediates, TCA cycle intermediates, and energy carrier molecules in 6-week-old *Atgl*^*flox/flox*^ [*n* = 4], *cAtgl*^*−/−*^[*n* = 4], and *cAtgl*^*−/−*^*Lpl*^*−/−*^ [*n* = 4] mice. TCA, tricarboxylic acid.

Loss of LpL and CD36 might be compensated by upregulation of another molecule that participates in lipid uptake into the heart. To determine this, we next used a big data approach, studying gene transcription changes in hearts with ATGL deficiency. Heart failure leads to a metabolic switch from FA to glucose associated with decreased expression of FA oxidation enzymes (28). To avoid the confounding effects of heart failure, we investigated *cAtgl*^*−/−*^ mice before the development of cardiac dysfunction, at 6 weeks. Gene expression of lipid uptake proteins (*Cd36*, *Fatp1*, and *Fatp5*), lipid catabolic genes (*Acsl1*, *Acadm*, and *PPARα*), and tricarboxylic acid cycle enzymes was decreased in *cAtgl*^*−/−*^ and *cAtgl*^*−/−*^*Lpl*^*−/−*^ hearts suggesting that uptake of exogenous lipids is not an important source of lipid accumulation (Fig. 6A). A number of genes important in lipid synthesis were unchanged including *Fasn* (Fig. 6A). Cell death was the most upregulated pathway, whereas mitochondrion and cellular oxidation processes were the most downregulated (Fig. 6B). Consistent with a decrease in mitochondrial function, metabolomic analysis showed a decrease in tricarboxylic acid cycle intermediates (citric acid, fumaric acid, and l-malic acid). Levels of glycolysis intermediates and energy carrier molecules were largely unchanged (Fig. 6C).

### Effects of ATGL deficiency on cardiac lipidomics

At 6 weeks, ATGL-deficient hearts have increased lipid accumulation (2) despite no impairment of EF (1). We first assessed the distribution of ceramides, which are associated with cardiac dysfunction (29, 30). Several long-chain ceramides—18:1/19:0, 15:2/22:0, and 16:2/18:0—were increased, although others—18:1/24:0 and 15:2/22:0—were decreased (supplemental Fig. S4). Other lipids including phosphoethanolamines, phosphocholines, and sphingomyelins also had some changes in their distribution. Thus, it was unclear whether a specific lipid species contributed to heart dysfunction.

### LDs accumulate in cultured cardiomyocytes

**Fig. 7:**
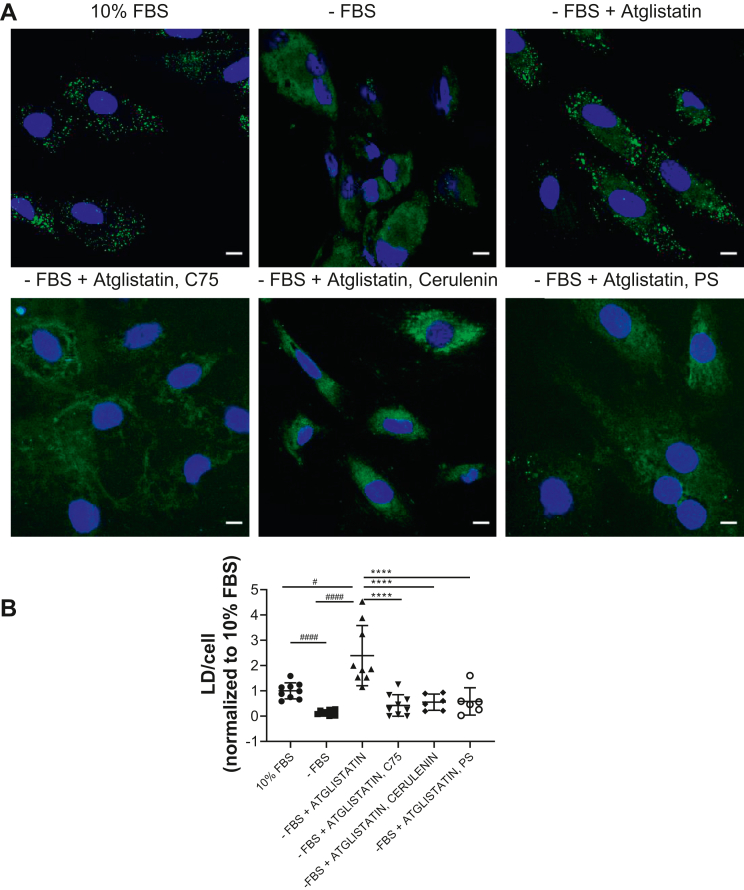
Cardiomyocyte cell culture examination of ATGL deficiency. A: Cells were grown in DMEM, 10% FBS, and incubated in DMEM without FBS for 24 h to deplete LDs. Those cells were then treated with atglistatin plus or minus FASN inhibitors C75, cerulenin, or platensimycin. B: Quantification. Statistical significance was determined by one-way ANOVA with Tukey multiple comparisons test; ∗*P* < 0.05, ∗∗*P* < 0.01, ∗∗∗*P* < 0.001, and ∗∗∗∗*P* < 0.0001 (significantly different to atglistatin treatment); ^#^*P* < 0.05, ^###^*P*< 0.001 (significantly different to 10% FBS treatment). The scale bars represent 20 μm.

Taken together, our in vivo data suggested that exogenous lipids do not cause lipid accumulation in ATGL deficiency. In contrast, normal cells cultured with FBS accumulate LDs that disappear when FBS is removed. To test this, we cultured H9c2(2-1) rat cardiomyocytes in media with and without lipids and inhibited ATGL using atglistatin (15). Cells cultured in DMEM supplemented with 10% FBS developed LDs, and an overnight incubation with FBS-free DMEM led to LD depletion. When we then inhibited ATGL in these LD-free cells, LDs accumulated despite the absence of FBS or any other extracellular lipid source (Fig. 7A, upper panels, green, quantified in Fig. 7B). To assess whether de novo FA synthesis was involved in this process, we inhibited FASN using three different inhibitors: C75, cerulenin and platensimycin streptomyces (Fig. 7A, lower panels, quantified in Fig. 7B). FASN inhibition markedly decreased LD accumulation in ATGL-inhibited cells.

### ATGL inhibition increases autophagy

**Fig. 8:**
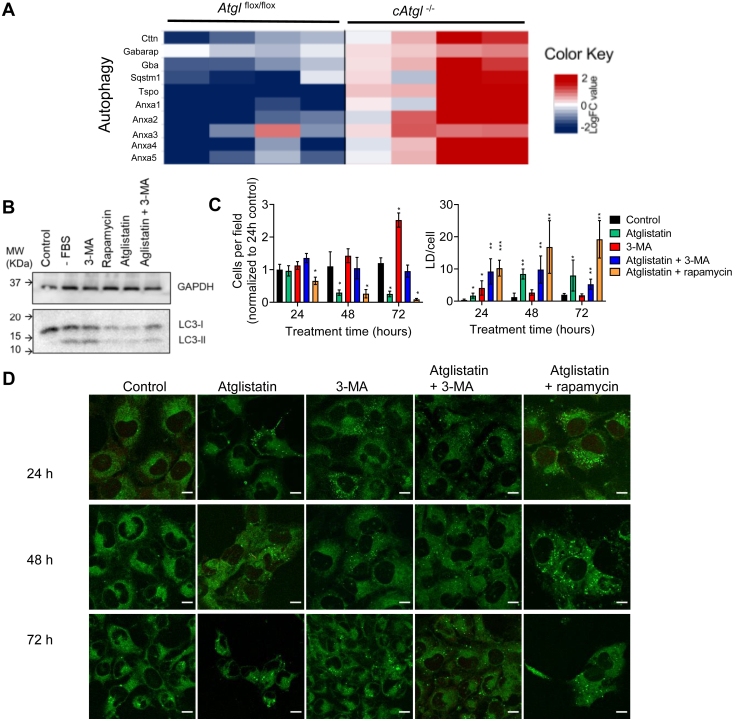
Autophagy induction in hearts from c*Atgl*^−/−^ mice. A: RNA-Seq analysis of changes between 6-week-old *Atgl*^*flox/flox*^ and *cAtgl*^*−/−*^ mice in expression of genes associated with autophagy regulation. AC16 cardiomyocyte survival. B: LC3 immunoblot of AC16 cells. C, left panel: Quantification of cells per field as a measure of survival overtime of AC16 cells incubated in DMEM without FBS treated with DBSO (control), atglistatin, 3-MA, atglistatin + 3-MA, and atglistatin + rapamycin. C, right panel: Quantification of LD content per cell. D: Representative confocal microscopy images. Cells were stained with BODIPY 493/503 to highlight LDs. Statistical significance was determined by unpaired *t*-tests; ∗*P* < 0.05, ∗∗*P* < 0.01, ∗∗∗*P* < 0.001, and ∗∗∗∗*P* < 0.0001. The scale bars represent 20 μm.

Our in vitro studies suggested that ATGL deficiency leads to TG accumulation because of defective TG lipolysis of exogenous and endogenous lipids. Our RNA-Seq data revealed that hearts from c*Atgl*^*−/−*^ mice had increased expression of several genes known to participate in the regulation of autophagy, including p62 (*Sqstm1*) and members of the annexin A family (31, 32) (Fig. 8A). To assess whether excessive, “maladaptive” autophagy induction has a role in heart dysfunction in the context of ATGL deficiency, we treated cardiomyocyte cell lines (AC16 and H9c2) with atglistatin and then switched the cells to FBS-free medium. Autophagy, as monitored by the appearance of LC3-II in an immunoblot, was induced by FBS-free medium but was further enhanced by treatment with atglistatin. Atglistatin induction of autophagy was equivalent to that found with rapamycin (an autophagy activator) treatment. 3-MA, a selective phosphoinositide 3-kinase (PI3K) inhibitor commonly used to block autophagosome biogenesis, strongly reduced autophagy in atglistatin-treated cells (Fig. 8B). Moreover, inhibition of autophagy in ATGL-knockdown FBS-deprived AC16 cardiomyocytes (over a 72 h timecourse) using 3-MA increased cell number and rescued AC16 cardiomyocytes treated with atglistatin. Conversely, cotreatment with 100 nM rapamycin did not improve viability (Fig. 8C, left panel, representative images are shown in Fig. 8D). In addition, we observed that inhibition of autophagy with 3-MA led to a further accumulation of LDs in atglistatin-treated cells (Fig. 8C, right panel).

The role of autophagy in the degradation of LDs (termed “lipophagy”) has been well established since its discovery in 2009 (33). To assess whether lipophagy specifically, rather than macroautophagy in general, is responsible for the toxicity observed in ATGL-inhibited cardiomyocytes, we used lalistat 1 to inhibit lysosomal lipase. As we had previously observed (Fig. 7), inhibition of ATGL in FBS-deprived cardiomyocytes resulted in increased cell death (≈80% after a 72 h treatment) (supplemental Fig. S5A, quantified in B) and LD accumulation (supplemental Fig. S5C). As expected, treatment with lalistat increased LD content, consistent with the established role of autophagy in LD degradation (33). However, in contrast to 3-MA, treatment with 1 μM lalistat 1 increased cell death (≈50% after a 72 h treatment) and failed to rescue ATGL-inhibited cardiomyocytes (supplemental Fig. S5A, quantified in B). These results suggest that, whereas lysosomal lipid hydrolysis may sustain cell viability in FBS-deprived cardiomyocytes, the products of that hydrolysis are unlikely to be responsible for the toxicity observed in ATGL-inhibited cells.

While ATGL deficiency leads to many cellular abnormalities including lipid accumulation and reduced FA oxidation, our data suggest that another toxicity is an excessive induction of autophagy.

## Discussion

Cardiac ATGL deficiency causes massive TG accumulation in the heart that leads to cardiac failure and death. ATGL deficiency in humans has no known effective therapy. We first tested whether dietary manipulation affected heart lipid accumulation, heart dysfunction, and mortality of mice with heart ATGL deficiency. This strategy has been suggested for humans, but with limited clinical outcome data (PMID: 9301368). Heart lipid accumulation and premature death occurred in mice eating either high- or low-fat diets. We then found that unlike other models of lipid toxicity (6, 7), deleting lipid uptake pathways did not rescue cardiac ATGL-deficient mice. Neither reducing TG lipolysis in the heart because of LpL KO nor reducing FA uptake by deletion of *Cd36* affected lipid accumulation or improved hearts in *Atgl* KO mice. Using cultured cardiomyocytes, we saw that ATGL inhibition led to marked LD accumulation even in the absence of an exogenous lipid source. This lipid accumulation was prevented by treatment with FASN inhibitors, suggesting that the accumulation can occur via de novo synthesis. Our data show that the toxicity in ATGL deficiency cannot be prevented by reducing cardiac lipid uptake.

A mutation of the *Atgl* gene in humans causes lipid accumulation, myopathy, and cardiomyopathy (5, 34). The clinical presentation of ATGL deficiency varies with some patients presenting early in life with exercise intolerance, palpitations, or chest pain (5, 35). Others present later in life (40+) with muscle weakness (5, 34). There are currently no established treatments for ATGL-deficient patients, although low-fat diets and PPARα receptor agonists have been used (11, 36). The reasons why skeletal muscle symptoms rather than cardiac symptoms predominate in humans are unclear but might reflect the relatively greater cardiac nutritional demands in the rapidly beating mouse heart. Our goal had been to define a method to treat ATGL deficiency by studying mice. In both mice and humans, ATGL catalyzes the rate-limiting step of LD TG hydrolysis (37, 38, 39).

We had hypothesized incorrectly that hindering lipid uptake would reduce LD accumulation and prevent cardiomyopathy. Rather, we found that even in the absence of exogenous lipids, ATGL knockdown led to LDs. Furthermore, the amount of lipid from the diet or in the culture media did not correlate with the degree of heart dysfunction or cardiomyocyte death. These data suggest that defective FA oxidation leads to lipid accumulation from either circulating or endogenously produced lipids. Although cardiomyocyte synthesis of FAs is relatively low, this locally produced TG accumulates likely because of reduced activation of PPARα. This conclusion is, however, a bit tenuous as a marked reduction in PPARα signaling was not found in inducible cardiomyocyte-specific *Atgl* KO (3). Likewise, our younger (6-week-old) cardiomyocyte-specific ATGL-deficient mice show a less dramatic decrease in the PPAR signaling pathway. In contrast to mice, hearts from ATGL-deficient patients requiring transplantation had increased PPARα and PPARγ gene expression (40). Together, these data suggest a dissociation between PPAR activation and the development of heart dysfunction and imply that another process such as lipid-induced mitochondrial dysfunction results from ATGL deficiency.

Our failure to find a reduction in lipid accumulation and an improvement in heart function with low-fat diets and *Cd36* and LpL deletion led us to study ATGL-deficient hearts in more detail. Greater lipophagy accompanies nutrient deprivation as likely occurs in ATGL-deficient hearts that are unable to use FAs. Our gene array data obtained from hearts of mice prior to their development of dysfunction pointed to activation of the autophagy pathway. Our experiments in cell culture showed that inhibition of autophagy prevented cell death. Moreover, we found that increased LDs are not toxic when autophagy is suppressed. Data in humans support a role for greater autophagy in ATGL-deficient muscles; biopsies from patients with neutral lipid storage disease with myopathy have more vacuoles that are positive for autophagy markers LC3, p62, and Beclin-1 (41).

Why should this autophagy be harmful? The role of autophagy in heart physiology and pathology remains poorly understood. Treatments aimed at inducing autophagy may be cardioprotective after ischemic insult (42, 43, 44, 45). In contrast, “maladaptive” autophagic flux can lead to excessive degradation and depletion of cytosolic components—including mitochondria—and cardiomyocyte death (46). In addition, with increased autophagy, released lipids such as ceramides—seen in our lipidomics analysis of 6-week-old mice—could lead to mitochondrial toxicity and decreased oxidative capacity (2), which would further deplete mitochondria and promote LD accumulation (29, 47, 48).

Aside from increased autophagy, ATGL deficiency leads to a number of metabolic/cellular abnormalities. Is it possible that autophagy adds to these other defects? Blocking autophagy, as we show in cultured cells, may prevent mitochondrial toxicity and muscle dysfunction in ATGL deficiency. Support for a pathologic role of autophagy was obtained using 3-MA, a PI3K inhibitor that also blocks autophagy, as well as other cellular processes mediated by PI3K (49, 50). Thus, while not ideal, 3-MA inhibition of autophagy in ATGL-deficient cells improved survival and is further evidence for the importance of this pathway in cellular dysfunction. We note that a genetic model that completely blocks the autophagy pathway leads to myocardial growth defects (51). Whether a partial block in this pathway could be a therapeutic approach remains to be tested. It should be noted that we have obtained no evidence that altering autophagy in vivo will reduce heart dysfunction, as the greater gene expression indicative of activation of this pathway might primarily reflect the energetic defects in these mice. Moreover, because our cellular studies were done in lipid-depleted serum, a condition unlike that in the mice, the possibility that loss of lipophagy might exacerbate heart failure must be considered.

In summary, we tested whether reduced lipid uptake rescues dysfunction in ATGL deficiency. Lipid accumulation and heart dysfunction in cardiac ATGL-deficient mice is not corrected by changes in diet. Unlike in other lipotoxicity models, reduced expression of either LpL or CD36 did not modify lipid accumulation or toxicity. We discovered that the lipid accumulation in ATGL-deficient cardiomyocytes can occur via de novo synthesis. ATGL-deficient cardiomyopathy is often presented as a model of lipid-induced heart dysfunction. As such, lessons learned from this model might be applicable to the much more common forms of cardiomyopathy associated with human obesity (52, 53, 54). While heart failure in patients with obesity and type 2 diabetes often associates with lipid accumulation, the source of that lipid might not be from circulating FAs or lipoproteins. Rather changes in cellular metabolism will drive TG accumulation and heart dysfunction via uptake of either FAs or substrates for de novo lipogenesis.

## Data Availability

All data are contained within the article.

## Supplemental Data

This article contains supplemental data.

## Conflict of Interest

A. E. M. is an employee of Ionis Pharmaceutical. All other authors declare that they have no conflicts of interest with the contents of this article.

## References

[1] Haemmerle G., Lass A., Zimmermann R., Gorkiewicz G., Meyer C., Rozman J. (2006). Defective lipolysis and altered energy metabolism in mice lacking adipose triglyceride lipase. Science.

[2] Haemmerle G., Moustafa T., Woelkart G., Büttner S., Schmidt A., van de Weijer T. (2011). ATGL-mediated fat catabolism regulates cardiac mitochondrial function via PPAR-α and PGC-1. Nat. Med..

[3] Kienesberger P.C., Pulinilkunnil T., Nagendran J., Young M.E., Bogner-Strauss J.G., Hackl H. (2013). Early structural and metabolic cardiac remodelling in response to inducible adipose triglyceride lipase ablation. Cardiovasc. Res..

[4] Natali A., Gastaldelli A., Camastra S., Baldi S., Quagliarini F., Minicocci I. (2013). Metabolic consequences of adipose triglyceride lipase deficiency in humans: an in vivo study in patients with neutral lipid storage disease with myopathy. J. Clin. Endocrinol. Metab..

[5] Zhang W., Wen B., Lu J., Zhao Y., Hong D., Zhao Z. (2019). Neutral lipid storage disease with myopathy in China: a large multicentric cohort study. Orphanet J. rare Dis..

[6] Duncan J.G., Bharadwaj K.G., Fong J.L., Mitra R., Sambandam N., Courtois M.R. (2010). Rescue of cardiomyopathy in peroxisome proliferator-activated receptor-alpha transgenic mice by deletion of lipoprotein lipase identifies sources of cardiac lipids and peroxisome proliferator-activated receptor-alpha activators. Circulation.

[7] Yang J., Sambandam N., Han X., Gross Richard W., Courtois M., Kovacs A. (2007). CD36 Deficiency Rescues Lipotoxic Cardiomyopathy. Circ. Res..

[8] Son N.-H., Basu D., Samovski D., Pietka T.A., Peche V.S., Willecke F. (2018). Endothelial cell CD36 optimizes tissue fatty acid uptake. J. Clin. Invest..

[9] Habets D.D.J., Coumans W.A., Voshol P.J., den Boer M.A.M., Febbraio M., Bonen A. (2007). AMPK-mediated increase in myocardial long-chain fatty acid uptake critically depends on sarcolemmal CD36. Biochem. Biophysical Res. Commun..

[10] Augustus A., Yagyu H Fau - Haemmerle G., Haemmerle G., Fau - Bensadoun A., Bensadoun A Fau - Vikramadithyan R.K., Vikramadithyan Rk (2004). Cardiac-specific knock-out of lipoprotein lipase alters plasma lipoprotein triglyceride metabolism and cardiac gene expression. J. Biol. Chem.

[11] Kakourou T., Drogari E., Christomanou H., Giannoulia A., Dacou-Voutetakis C. (1997). Neutral lipid storage disease--response to dietary intervention. Arch. Dis. Child..

[12] Zhou W., Simpson P.J., McFadden J.M., Townsend C.A., Medghalchi S.M., Vadlamudi A. (2003). Fatty acid synthase inhibition triggers apoptosis during S phase in human cancer cells. Cancer Res..

[13] Wu M., Singh S.B., Wang J., Chung C.C., Salituro G., Karanam B.V. (2011). Antidiabetic and antisteatotic effects of the selective fatty acid synthase (FAS) inhibitor platensimycin in mouse models of diabetes. Proc. Natl. Acad. Sci. U. S. A..

[14] Thupari J.N., Pinn M.L., Kuhajda F.P. (2001). Fatty acid synthase inhibition in human breast cancer cells leads to malonyl-CoA-induced inhibition of fatty acid oxidation and cytotoxicity. Biochem. Biophysi. Res. Commun..

[15] Mayer N., Schweiger M., Fuchs E., Migglautsch A.K., Doler C., Grabner G.F. (2020). Structure-activity relationship studies for the development of inhibitors of murine adipose triglyceride lipase (ATGL). Bioorg. Med. Chem..

[16] Folch J., Lees M., Sloane Stanley G.H. (1957). A simple method for the isolation and purification of total lipides from animal tissues. J. Biol. Chem..

[17] Love M.I., Huber W., Anders S. (2014). Moderated estimation of fold change and dispersion for RNA-seq data with DESeq2. Genome Biol..

[18] Pacold M.E., Brimacombe K.R., Chan S.H., Rohde J.M., Lewis C.A., Swier L.J. (2016). A PHGDH inhibitor reveals coordination of serine synthesis and one-carbon unit fate. Nat. Chem. Biol..

[19] Chen W.W., Freinkman E., Wang T., Birsoy K., Sabatini D.M. (2016). Absolute quantification of matrix metabolites reveals the dynamics of mitochondrial metabolism. Cell.

[20] Simón-Manso Y., Lowenthal M.S., Kilpatrick L.E., Sampson M.L., Telu K.H., Rudnick P.A. (2013). Metabolite profiling of a NIST standard reference material for human plasma (SRM 1950): GC-MS, LC-MS, NMR, and clinical laboratory analyses, libraries, and web-based resources. Anal Chem..

[21] Jones E., Oliphant T., Peterson P. (2001).

[22] van Beijnum J.R., Rousch M., Castermans K., van der Linden E., Griffioen A.W. (2008). Isolation of endothelial cells from fresh tissues. Nat. Protoc..

[23] Cabodevilla A.G., Tang S., Lee S., Mullick A.E., Aleman J.O., Hussain M.M. (2021). Eruptive xanthoma model reveals endothelial cells internalize and metabolize chylomicrons, leading to extravascular triglyceride accumulation. J. Clin. Invest..

[24] Fischer A.W., Jaeckstein M.Y., Gottschling K., Heine M., Sass F., Mangels N. (2021). Lysosomal lipoprotein processing in endothelial cells stimulates adipose tissue thermogenic adaptation. Cell Metab.

[25] Sung M.M., Byrne N.J., Kim T.T., Levasseur J., Masson G., Boisvenue J.J. (2017). Cardiomyocyte-specific ablation of CD36 accelerates the progression from compensated cardiac hypertrophy to heart failure. Am. J. Physiol. Heart Circ. Physiol..

[26] Irie H., Krukenkamp I.B., Brinkmann J.F.F., Gaudette G.R., Saltman A.E., Jou W. (2003). Myocardial recovery from ischemia is impaired in CD36-null mice and restored by myocyte CD36 expression or medium-chain fatty acids. Proc. Natl. Acad. Sci. U. S. A..

[27] Trent C.M., Yu S., Hu Y., Skoller N., Huggins L.A., Homma S. (2014). Lipoprotein lipase activity is required for cardiac lipid droplet production. J. Lipid Res..

[28] Barger P.M., Kelly D.P. (2000). PPAR Signaling in the Control of Cardiac Energy Metabolism. Trends Cardiovasc. Med..

[29] Law B.A., Liao X., Moore K.S., Southard A., Roddy P., Ji R. (2018). Lipotoxic very-long-chain ceramides cause mitochondrial dysfunction, oxidative stress, and cell death in cardiomyocytes. FASEB J..

[30] Green C.D., Maceyka M., Cowart L.A., Spiegel S. (2021). Sphingolipids in metabolic disease: the good, the bad, and the unknown. Cell Metab..

[31] Xi Y., Ju R., Wang Y. (2020). Roles of Annexin A protein family in autophagy regulation and therapy. Biomed. Pharmacother..

[32] Moreau K., Ghislat G., Hochfeld W., Renna M., Zavodszky E., Runwal G. (2015). Transcriptional regulation of Annexin A2 promotes starvation-induced autophagy. Nat. Commun..

[33] Singh R., Kaushik S., Wang Y., Xiang Y., Novak I., Komatsu M. (2009). Autophagy regulates lipid metabolism. Nature.

[34] Shi J., Qu Q., Liu H., Zhang Y., Cui W., Chen P. (2021). Case Report: PNPLA2 Gene Complex Heterozygous Mutation Leading to Neutral Lipid Storage Disease With Myopathy. Front. Integr. Neurosci..

[35] Zheng S., Liao W. (2018). Novel PNPLA2 gene mutation in a child causing neutral lipid storage disease with myopathy. BMC Med. Genet..

[36] van de Weijer T., Havekes B., Bilet L., Hoeks J., Sparks L., Bosma M. (2013). Effects of bezafibrate treatment in a patient and a carrier with mutations in the PNPLA2 gene, causing neutral lipid storage disease with myopathy. Circ. Res..

[37] Zimmermann R., Strauss J.G., Haemmerle G., Schoiswohl G., Birner-Gruenberger R., Riederer M. (2004). Fat mobilization in adipose tissue is promoted by adipose triglyceride lipase. Science.

[38] Villena J.A., Roy S., Sarkadi-Nagy E., Kim K.H., Sul H.S. (2004). Desnutrin, an adipocyte gene encoding a novel patatin domain-containing protein, is induced by fasting and glucocorticoids: ectopic expression of desnutrin increases triglyceride hydrolysis. J. Biol. Chem..

[39] Jenkins C.M., Mancuso D.J., Yan W., Sims H.F., Gibson B., Gross R.W. (2004). Identification, cloning, expression, and purification of three novel human calcium-independent phospholipase A2 family members possessing triacylglycerol lipase and acylglycerol transacylase activities. J. Biol. Chem..

[40] Hirano K.-i., Tanaka T., Ikeda Y., Yamaguchi S., Zaima N., Kobayashi K. (2014). Genetic mutations in adipose triglyceride lipase and myocardial up-regulation of peroxisome proliferated activated receptor-γ in patients with triglyceride deposit cardiomyovasculopathy. Biochem. Biophys. Res. Commun..

[41] Hong D., Zheng J., Xin L., Xiang Y., Luan X., Cao L. (2019). Clinical findings and autophagic pathology in neutral lipid storage disease with myopathy. Clin. Neuropathol..

[42] Ikeda Y., Shirakabe A., Maejima Y., Zhai P., Sciarretta S., Toli J. (2015). Endogenous Drp1 mediates mitochondrial autophagy and protects the heart against energy stress. Circ. Res..

[43] Sciarretta S., Zhai P., Shao D., Maejima Y., Robbins J., Volpe M. (2012). Rheb is a critical regulator of autophagy during myocardial ischemia: pathophysiological implications in obesity and metabolic syndrome. Circulation.

[44] Andres A.M., Tucker K.C., Thomas A., Taylor D.J., Sengstock D., Jahania S.M. (2017). Mitophagy and mitochondrial biogenesis in atrial tissue of patients undergoing heart surgery with cardiopulmonary bypass. JCI insight.

[45] Schiattarella G.G., Hill J.A. (2016). Therapeutic targeting of autophagy in cardiovascular disease. J. Mol. Cell Cardiol..

[46] Zhu H., Tannous P., Johnstone J.L., Kong Y., Shelton J.M., Richardson J.A. (2007 Jul). Cardiac autophagy is a maladaptive response to hemodynamic stress. J. Clin. Invest..

[47] Bekhite M., González-Delgado A., Hübner S., Haxhikadrija P., Kretzschmar T., Müller T. (2021). The role of ceramide accumulation in human induced pluripotent stem cell-derived cardiomyocytes on mitochondrial oxidative stress and mitophagy. Free Radic. Biol. Med..

[48] Park T.-S., Hu Y., Noh H.-L., Drosatos K., Okajima K., Buchanan J. (2008). Ceramide is a cardiotoxin in lipotoxic cardiomyopathy. J. Lipid Res..

[49] Michl J., Silverstein S.C. (1978). Role of macrophage receptors in the ingestion phase of phagocytosis. Birth Defects. Orig Artic. Ser..

[50] Yang Y.-p., Hu L.-f., Zheng H.-f., Mao C.-j., Hu W.-d., Xiong K.-p. (2013). Application and interpretation of current autophagy inhibitors and activators. Acta Pharmacol. Sin..

[51] Kaizuka T., Mizushima N. (2015). Atg13 is essential for autophagy and cardiac development in mice. Mol. Cell Biol..

[52] Kenchaiah S., Evans J.C., Levy D., Wilson P.W., Benjamin E.J., Larson M.G. (2002). Obesity and the risk of heart failure. New Engl. J. Med..

[53] Alpert M.A., Lavie C.J., Agrawal H., Aggarwal K.B., Kumar S.A. (2014). Obesity and heart failure: epidemiology, pathophysiology, clinical manifestations, and management. Transl. Res..

[54] Wong C., Marwick T.H. (2007). Obesity cardiomyopathy: pathogenesis and pathophysiology. Nat. Clin. Pract. Cardiovasc. Med..

