# Supplementary material for: Blocking Lipid Uptake Pathways Does not Prevent Toxicity in Adipose Triglyceride Lipase (ATGL) Deficiency
Source: J Lipid Res. 2022 Sep 15;63(11):100274. doi: 10.1016/j.jlr.2022.100274 (PMC9618837; doi:10.1016/j.jlr.2022.100274)
Supplement: Supplementary Data [file mmc1.docx]

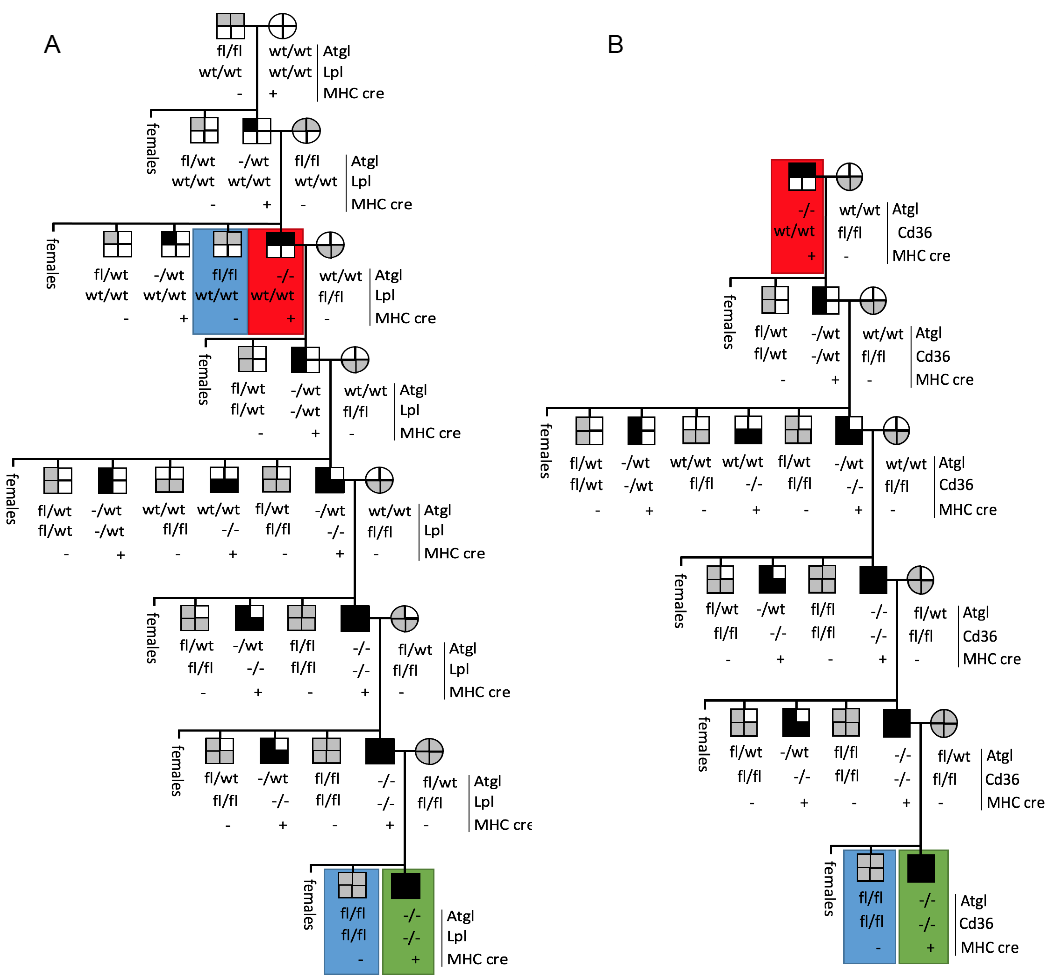


**Supplementary Figure 1. Breeding Scheme.** (A) Breeding schematic for the generation of *Atgl/Lpl* cardiomyocyte specific double knockout mice from *Atgl^flox/flox^* mice and MHC-Cre expressing mice all on a C57BL/6 background. Male mice are represented by squares and female mice by circles. White fill indicates wild type allele, grey fill indicates floxed allele without cre expression, and black fill indicates floxed allele in mice positive for cre expression. Experimental genotypes indicated with blue, red and green boxes. (B) Breeding schematic for the generation of *Atgl/Cd36* cardiomyocyte specific double knockout mice from *cAtgl^-/-^* mice and *Cd36^flox/flox^* mice all on a C57BL/6 background.

**
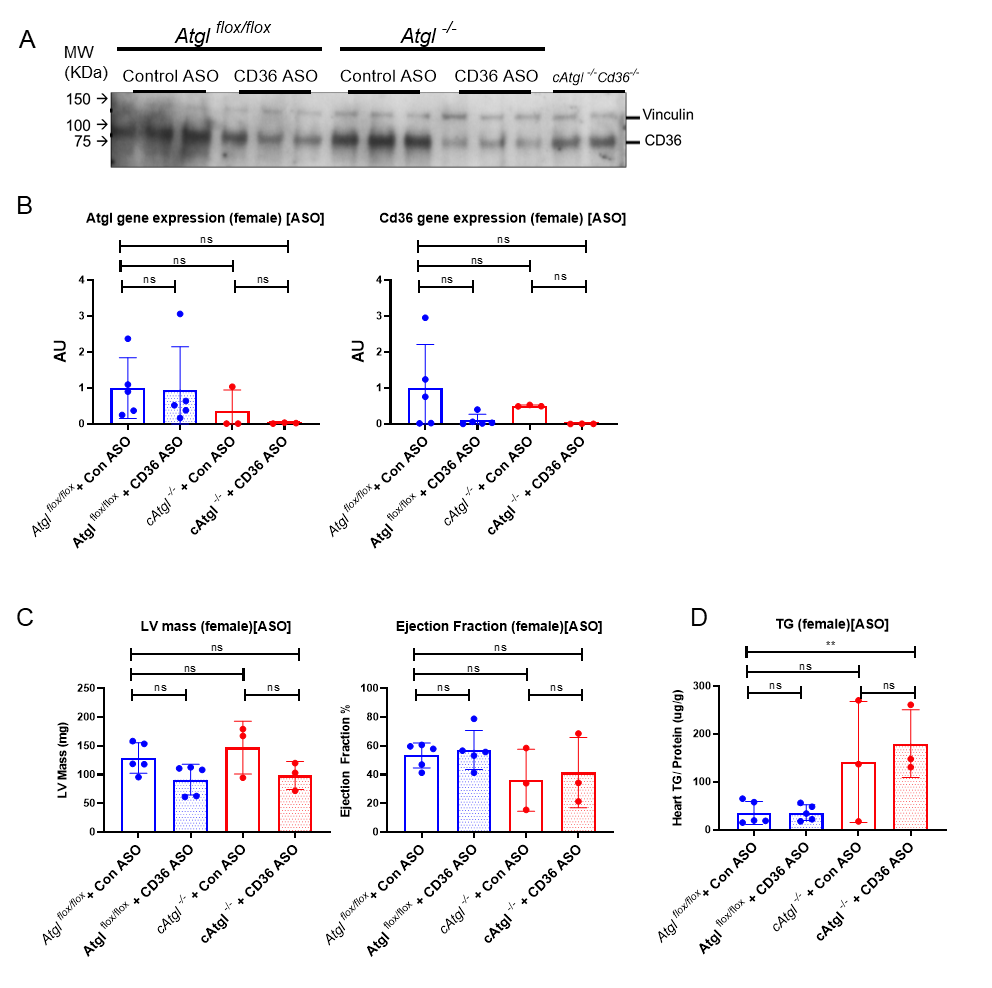
**

**Supplementary Figure 2. Heart function and lipid content of CD36 ASO treated *cAtgl^-/-^* mice.** (A)Experimental protocol for CD36 ASO or Control ASO treatment of *cAtgl^-/-^* mice. (B) *Atgl* and *Cd36* gene expression and CD36 protein changes in *Atgl^flox/flox^* and *cAtgl^-/-^* treated with either the control ASO or the CD36 ASO in female mice (*Atgl^flox/flox^* + Con ASO [n= 5]; *Atgl^flox/flox^* + CD36 ASO [n= 5]; *cAtgl^-/-^* + Con ASO[n= 3]; *cAtgl^-/-^* + CD36 ASO[n= 3]. (C) Echocardiography analysis of changes in LV mass and EF in the 4 treatment groups of female mice (*Atgl^flox/flox^* + Con ASO [n= 5]; *Atgl^flox/flox^* + CD36 ASO [n= 5]; *cAtgl^-/-^* + Con ASO[n= 3]; *cAtgl^-/-^* + CD36 ASO[n= 3]). (E) Heart TG changes from female mice of the 4 treatment (*Atgl^flox/flox^* + Con ASO [n= 5]; *Atgl^flox/flox^* + CD36 ASO [n= 5]; *cAtgl^-/-^* + Con ASO[n= 3]; *cAtgl^-/-^* + CD36 ASO[n= 3]). Statistical significance determined by 1-way ANOVA with Tukey’s multiple comparisons test; *P < 0.05, **P < 0.01, ***P < 0.001 and ****P < 0.0001.

**A**

**B**

**Supplementary Figure 3. Survival in *cAtgl^-/-^Lpl^-/-^* with CD36 ASO treatment.** Kaplan-Meier plot showing the cumulative survival of male *Atgl^flox/flox^Lpl^flox/flox^* + CON ASO (n= 2), *Atgl^flox/flox^Lpl^flox/flox^* + CD36 ASO (n= 2), *cAtgl^-/-^Lpl^-/-^* + CON ASO (n= 8), and *cAtgl^-/-^Lpl^-/-^* + CD36 ASO (n= 3) mice over 20 weeks. CD36 ASO treatment did not improve survival of *cAtgl^-/-^Lpl^-/-^* mice. Similarly, (B) Kaplan-Meier plot showing the cumulative survival of female *Atgl^flox/flox^Lpl^flox/flox^* + CON ASO (n= 3), *Atgl^flox/flox^Lpl^flox/flox^* + CD36 ASO (n= 5), *cAtgl^-/-^Lpl^-/-^* + CON ASO (n= 2), and *cAtgl^-/-^Lpl^-/-^* + CD36 ASO (n= 4) mice over 20 weeks showed no improvement.


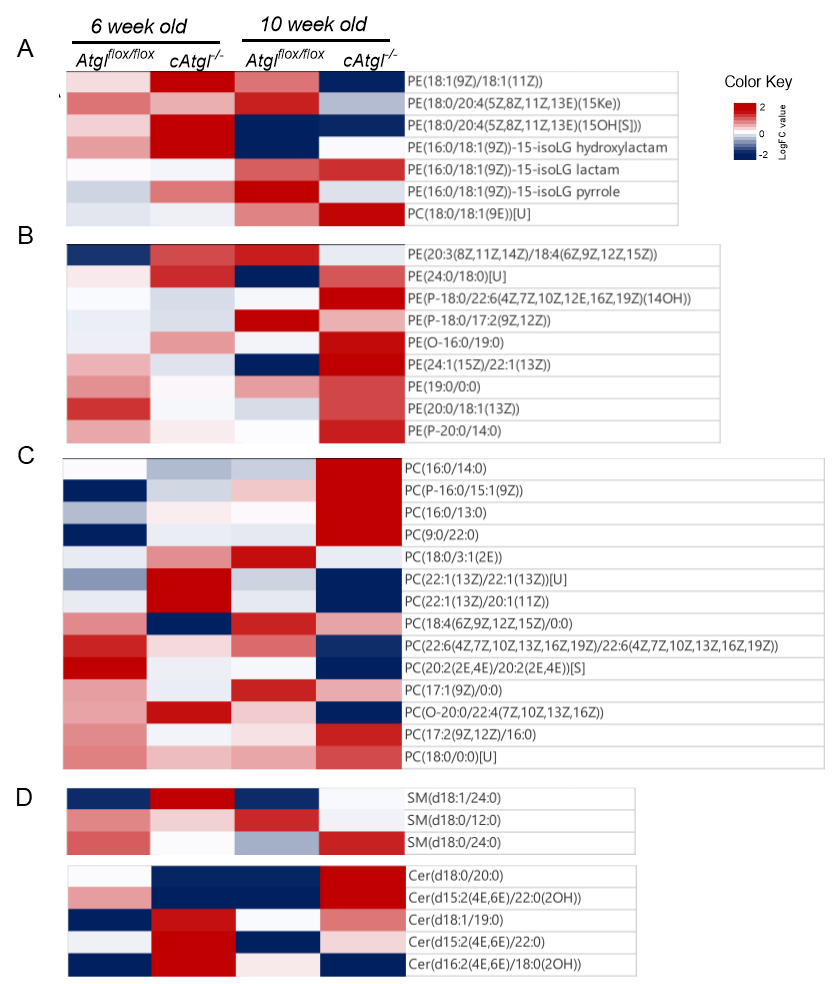


**Supplementary Figure 4. Lipidomics of young and adult *cAtgl^-/-^* mice**. Lipidomic analysis of 6 week old *Atgl^flox/flox^* [n=5] and *cAtgl^-/-^* [n=5] mice compared to their 10 week old *Atgl^flox/flox^* [n=4] and *cAtgl^-/-^* [n=4] counterparts with a focus on (A-C) PE/PC, (D) SM, and (E) Cer.


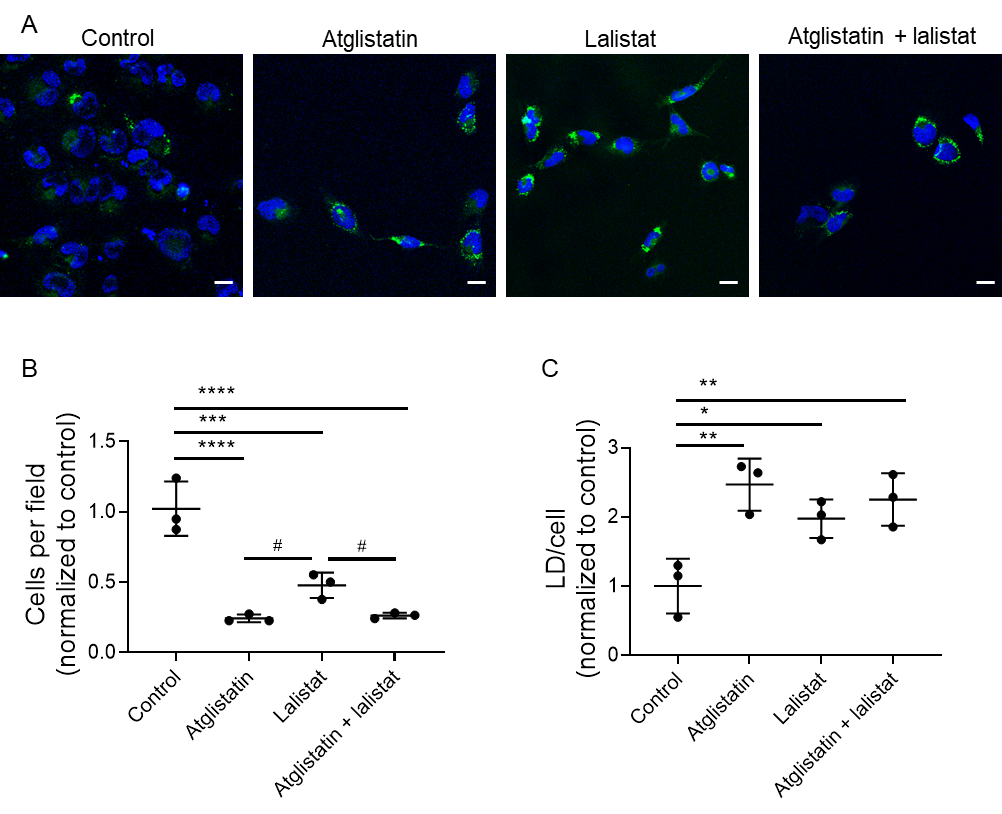


**Supplementary Figure 5. Inhibition of lipophagy with lalistat does not rescue ATGL-inhibited cardiomyocytes.** A. AC16 cardiomyocytes were deprived of serum overnight, then switched to FBS-free medium in the absence (control) or presence of atglistatin, lalistat, or both inhibitors simultaneously. After 72hs, cells were fixed and stained with BODIPY 493-504 (green) to label LD, and DAPI (blue) to highlight nuclei. Scale bars: 20μm. B. Quantification of cell viability. C. Quantification of LD content. Statistical significance determined by 1-way ANOVA with Tukey’s multiple comparisons test. *p<0.05, **p<0.01, ***p<0.001, ****p<0.0001 (significantly different to control). #p<0.05 (significantly different to lalistat).
